# Supplementary material for: Analysis of the Genetic Parameters for Dairy Linear Appraisal and Zoometric Traits: A Tool to Enhance the Applicability of Murciano-Granadina Goats Major Areas Evaluation System
Source: Animals (Basel). 2023 Mar 21;13(6):1114. doi: 10.3390/ani13061114 (PMC10044043; doi:10.3390/ani13061114)
Supplement: Supplementary file 1 [file animals-13-01114-s001.zip › Figures S1 to S27.pdf]

Figure S1: Graphical depictions of the scale for stature in Murciano-Granadina does for dairy purpose-related zoometric assessment.

Stature

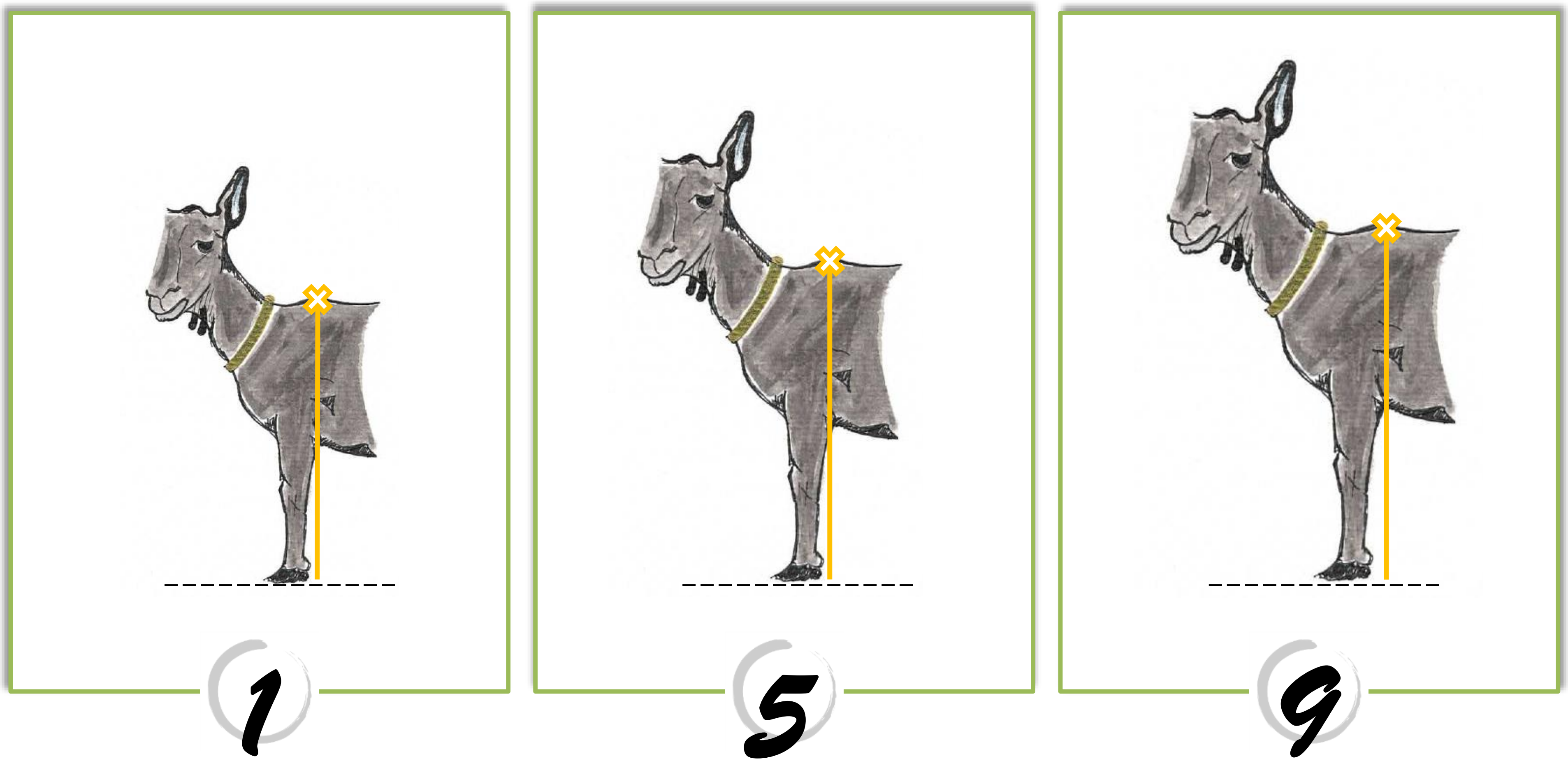

Measure unit	Score								
	1	2	3	4	5	6	7	8	9
cm	62	64	66	68	70	72	74	76	78

The scale translation from LAS scores to traditional zoometric measurements in centimeters, as well as optimal values following the premises described in the breed standard for both phenotype collection methods, are provided below the depictions.

Figure S2: Graphical depictions of the scale for chest width in Murciano-Granadina does for dairy purpose-related zoometric assessment.

Chest width

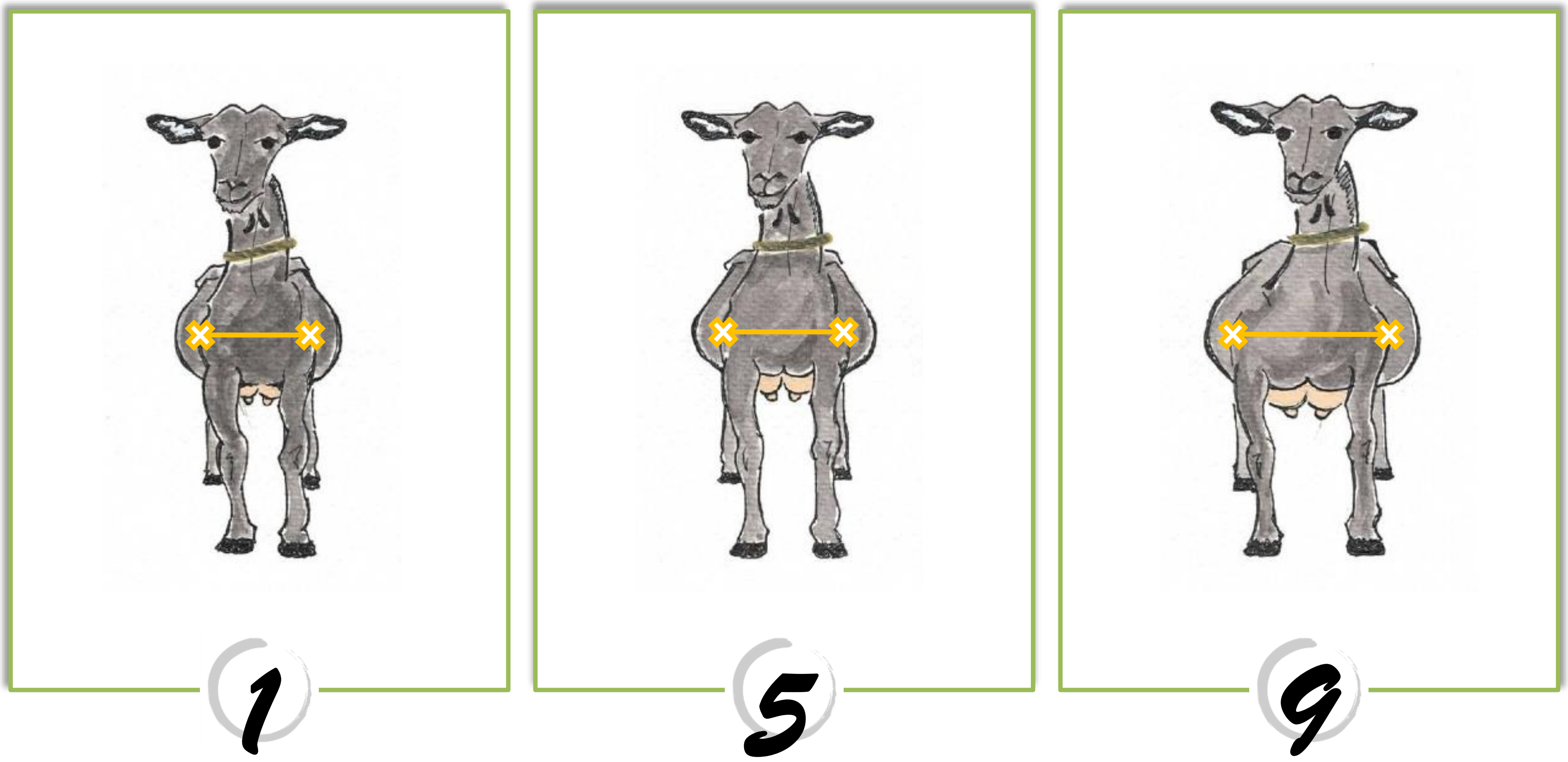

Measure unit	Score								
	1	2	3	4	5	6	7	8	9
cm	15	16	17	18	19	20	21	22	23

The scale translation from LAS scores to traditional zoometric measurements in centimeters, as well as optimal values following the premises described in the breed standard for both phenotype collection methods, are provided below the depictions.

Figure S3: Graphical depictions of the scale for body depth in Murciano-Granadina does for dairy purpose-related zoometric assessment.

Body depth

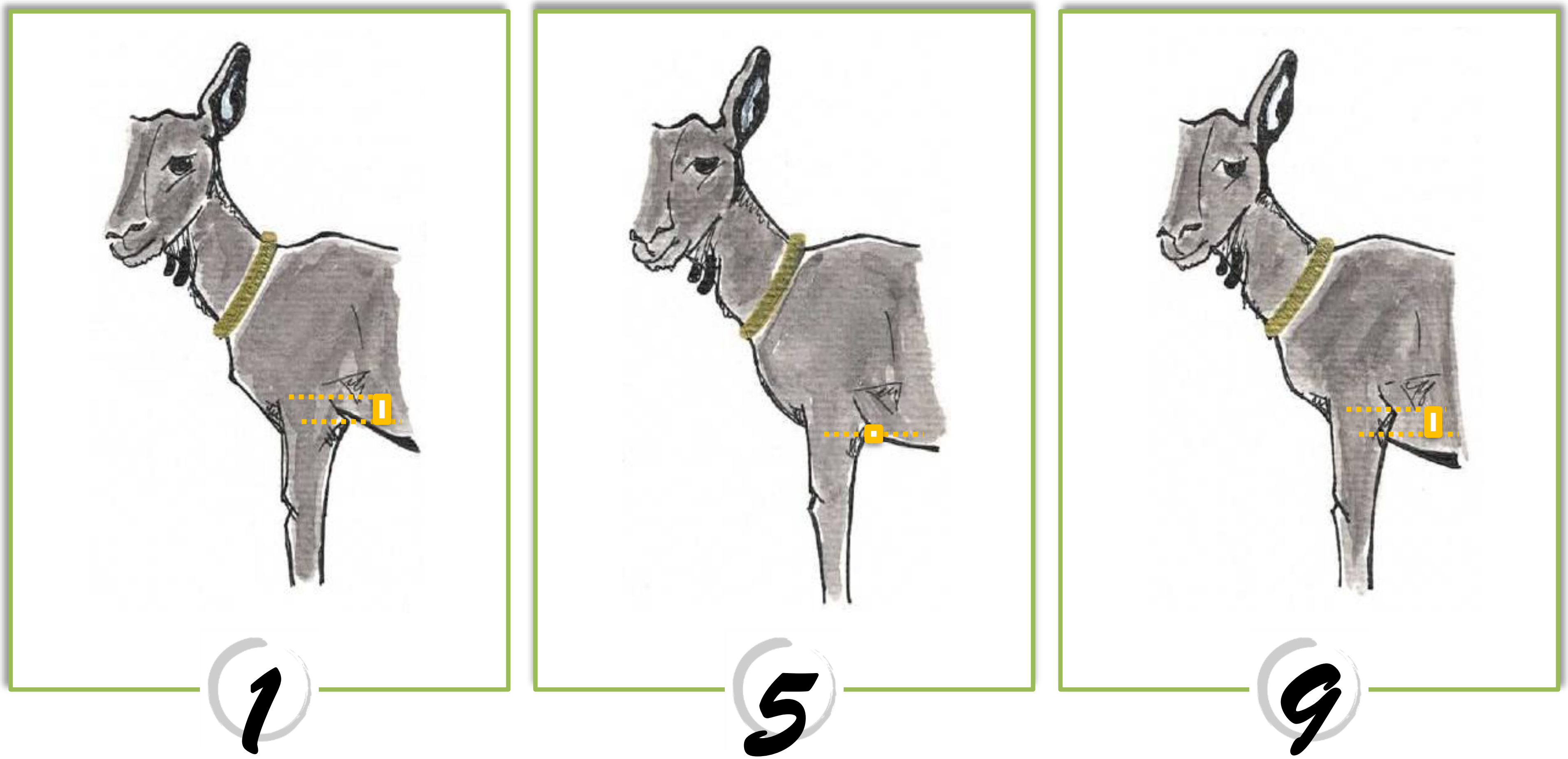

Score		
1	5	9
Shallow	Intermediate	Extremely deep

The scale translation from LAS scores to traditional zoometric measurements in centimeters, as well as optimal values following the premises described in the breed standard for both phenotype collection methods, are provided below the depictions.

Figure S4: Graphical depictions of the scale for rump width in Murciano-Granadina does for dairy purpose-related zoometric assessment.

Rump width

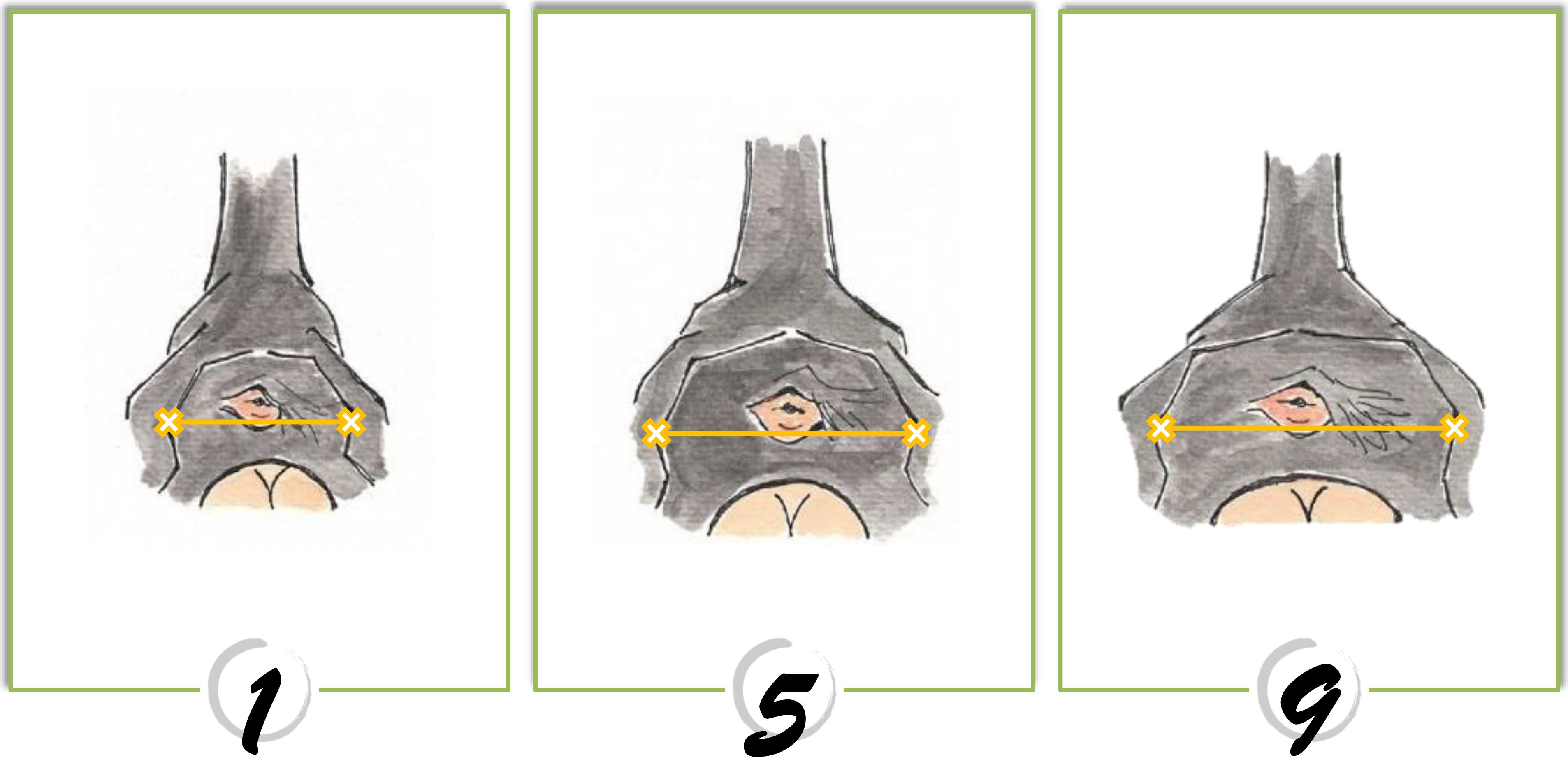

Measure unit	Score								
	1	2	3	4	5	6	7	8	9
cm	13	14	15	16	17	18	19	20	21

The scale translation from LAS scores to traditional zoometric measurements in centimeters, as well as optimal values following the premises described in the breed standard for both phenotype collection methods, are provided below the depictions.

Figure S5: Graphical depictions of the scale for rump angle in Murciano-Granadina does for dairy purpose-related zoometric assessment.

Rump angle

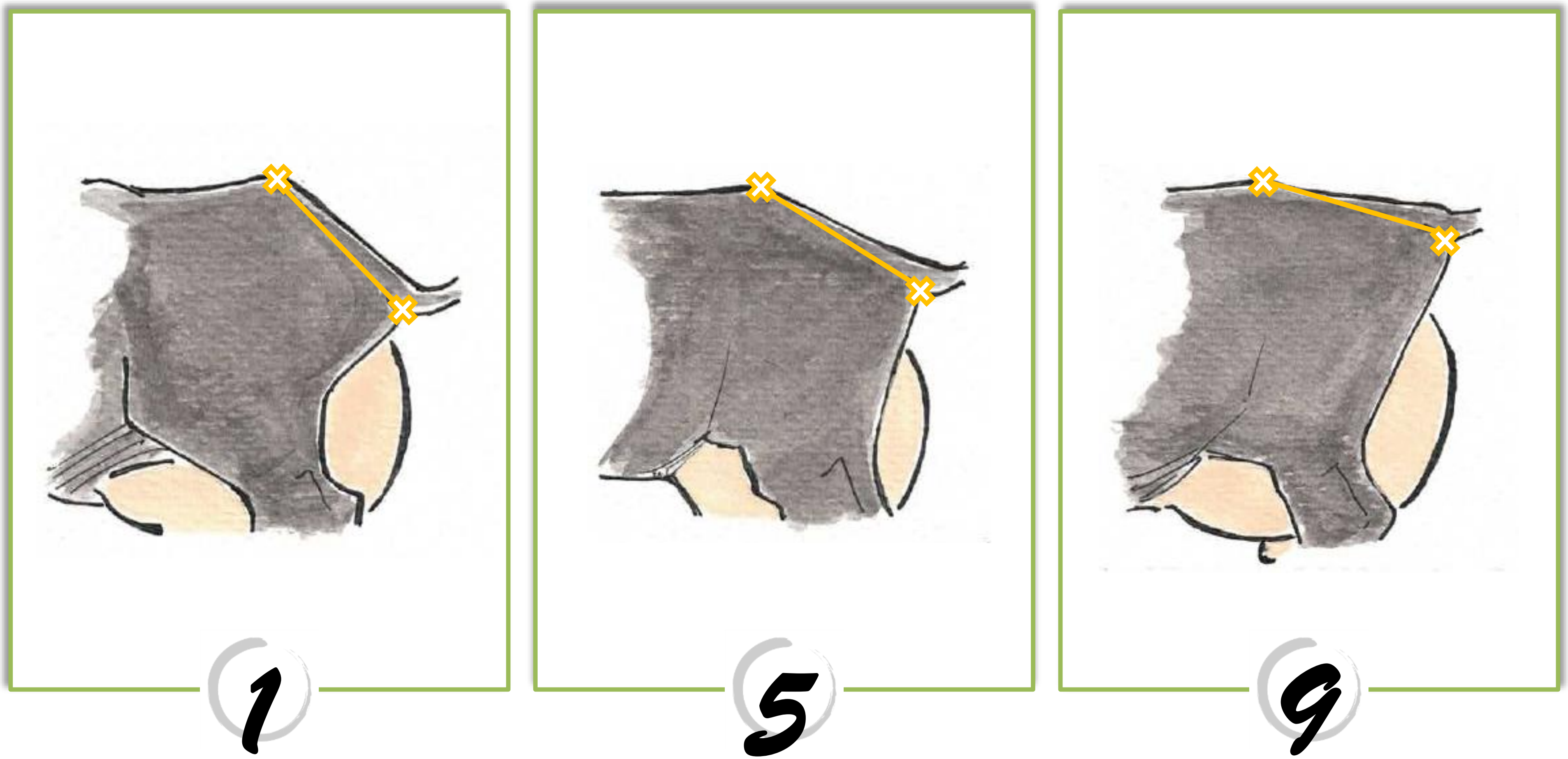

Measure unit	Score								
	1	2	3	4	5	6	7	8	9
cm	55	52	49	46	43	40	37	34	31

The scale translation from LAS scores to traditional zoometric measurements in centimeters, as well as optimal values following the premises described in the breed standard for both phenotype collection methods, are provided below the depictions.

Figure S6: Graphical depictions of the scale for angulosity in Murciano-Granadina does for dairy purpose-related zoometric assessment.

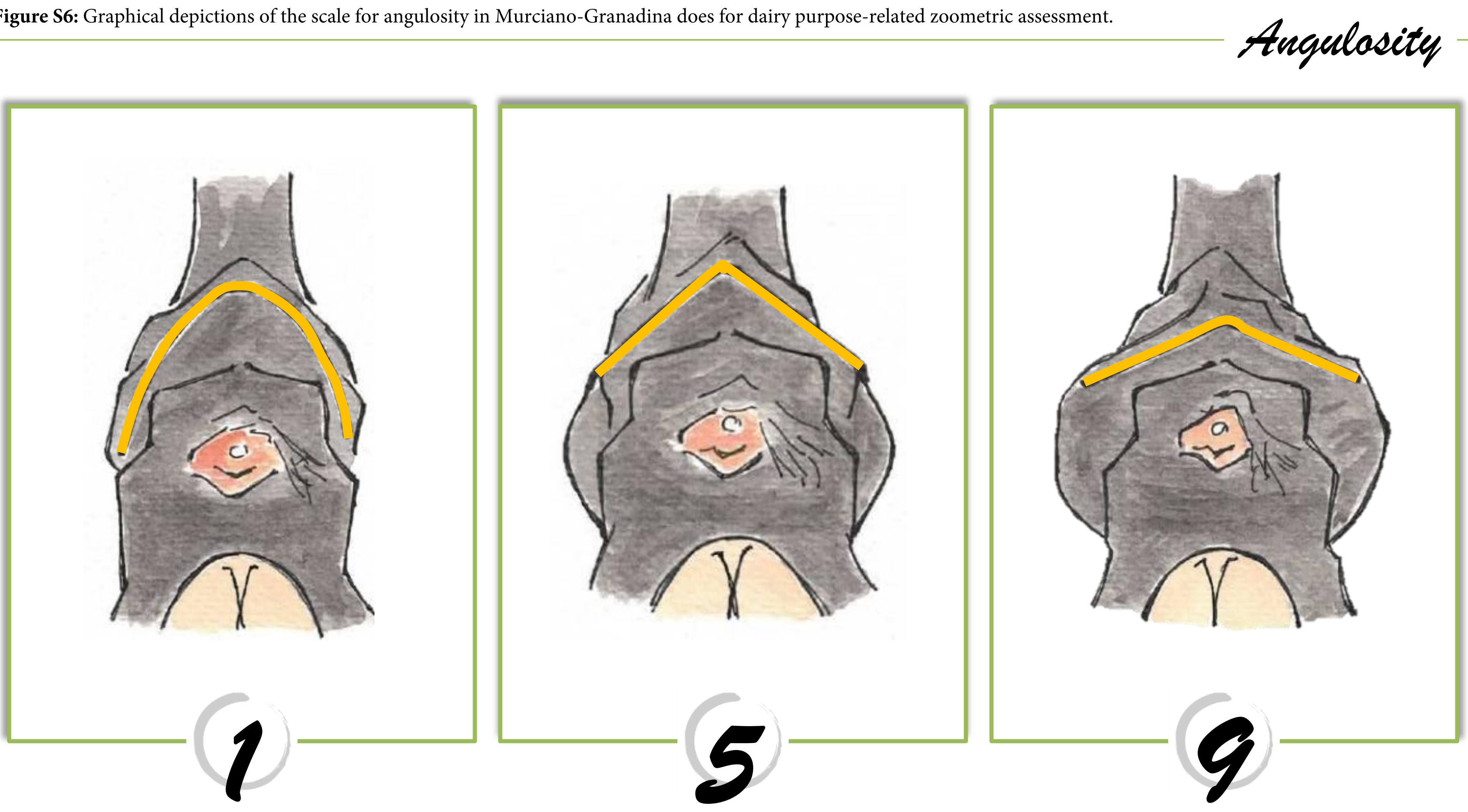

Score		
1	5	9
Rough extremity	Intermediate	Angulous extremity

The scale translation from LAS scores to traditional zoometric measurements in centimeters, as well as optimal values following the premises described in the breed standard for both phenotype collection methods, are provided below the depictions.

Figure S7: Graphical depictions of the scale for bone quality in Murciano-Granadina does for dairy purpose-related zoometric assessment.

Bone quality

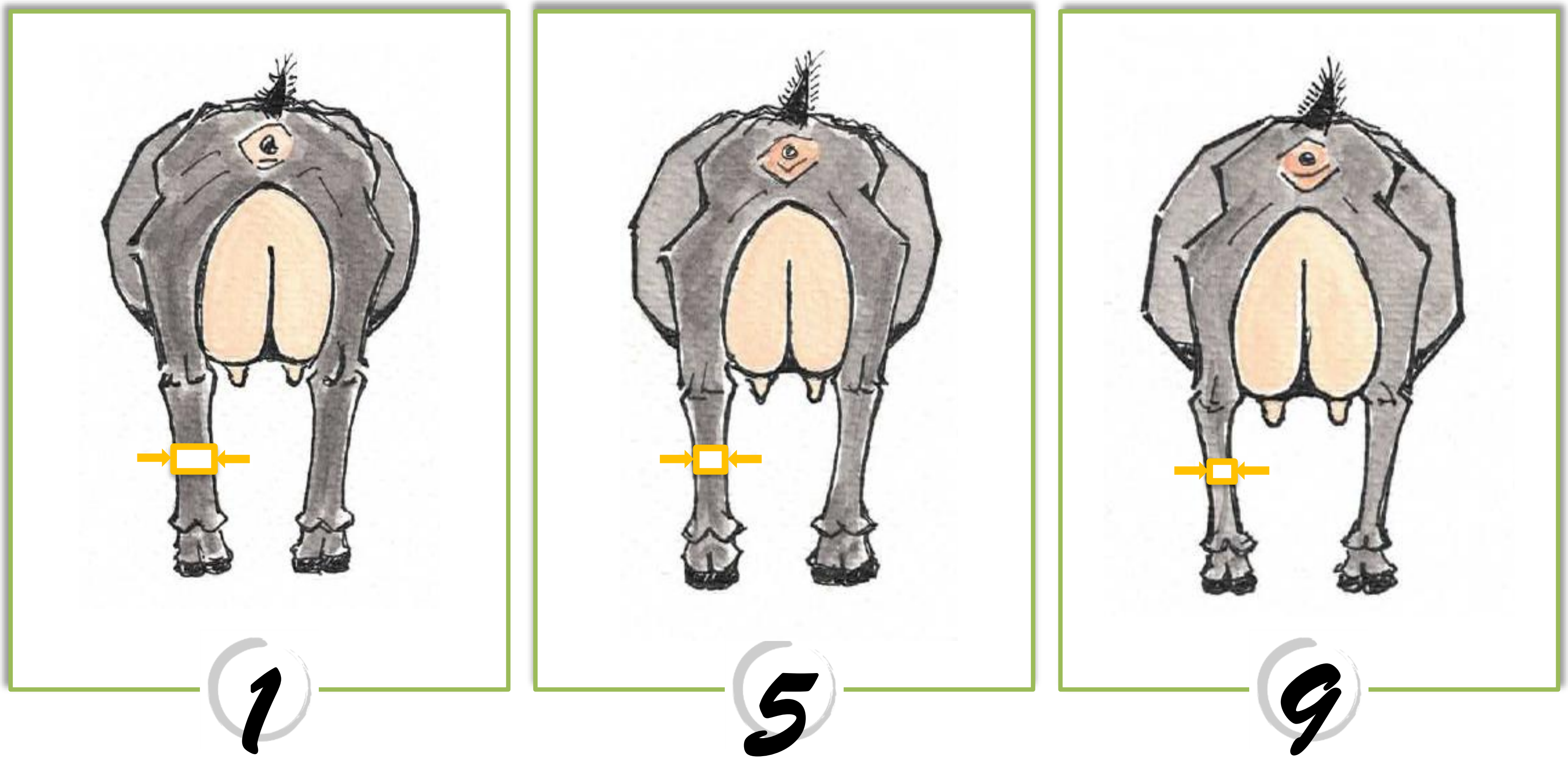

Score		
1	5	9
Round and rough bones	Intermediate	Flat and neat bones

The scale translation from LAS scores to traditional zoometric measurements in centimeters, as well as optimal values following the premises described in the breed standard for both phenotype collection methods, are provided below the depictions.

Figure S8: Graphical depictions of the scale for anterior insertion in Murciano-Granadina does for dairy purpose-related zoometric assessment.

Anterior insertion

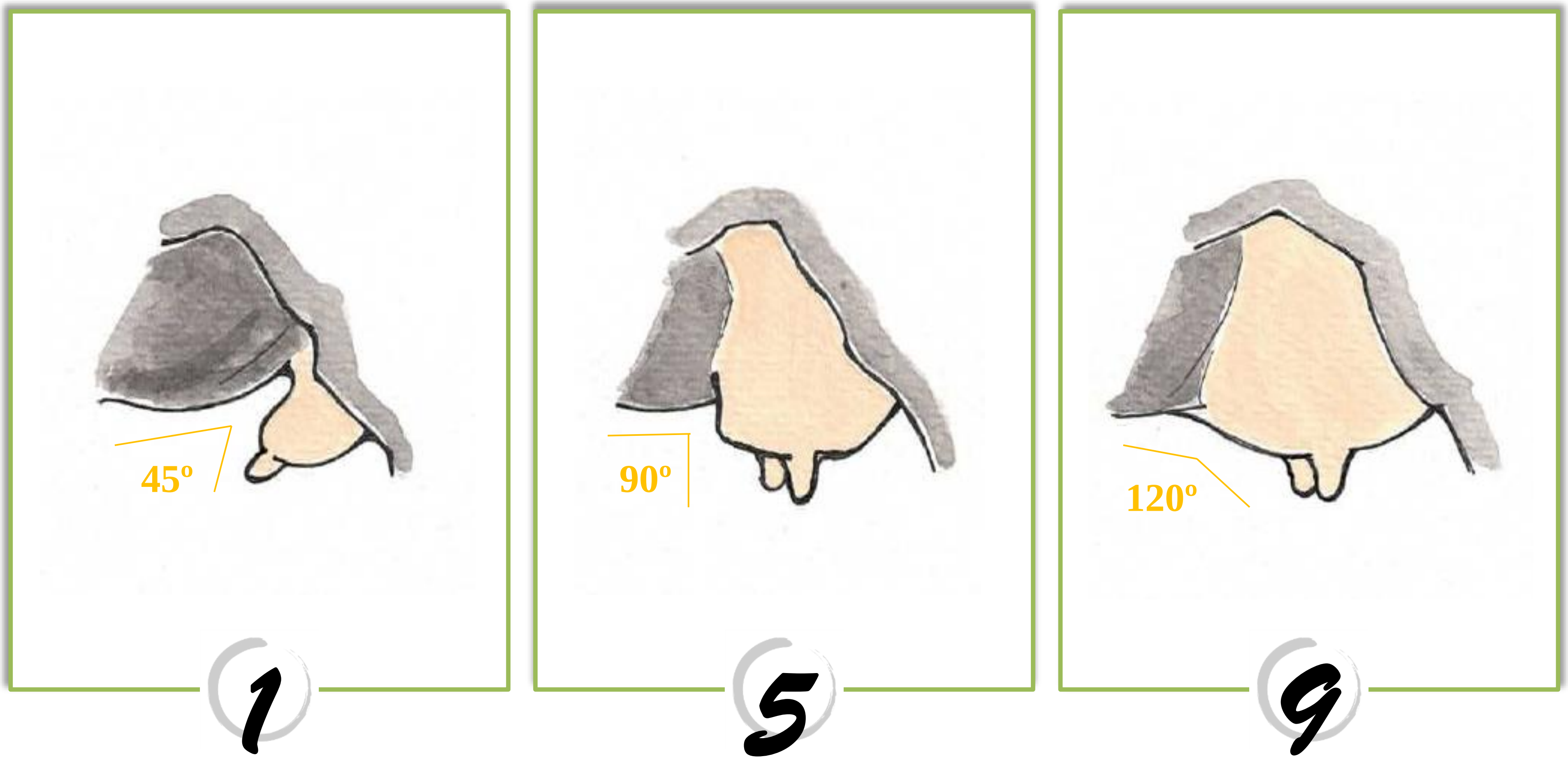

Score		
1	5	9
Weak	Intermediate	Strong

The scale translation from LAS scores to traditional zoometric measurements in centimeters, as well as optimal values following the premises described in the breed standard for both phenotype collection methods, are provided below the depictions.

Figure S9: Graphical depictions of the scale for rear insertion height in Murciano-Granadina does for dairy purpose-related zoometric assessment.

Rear insertion height

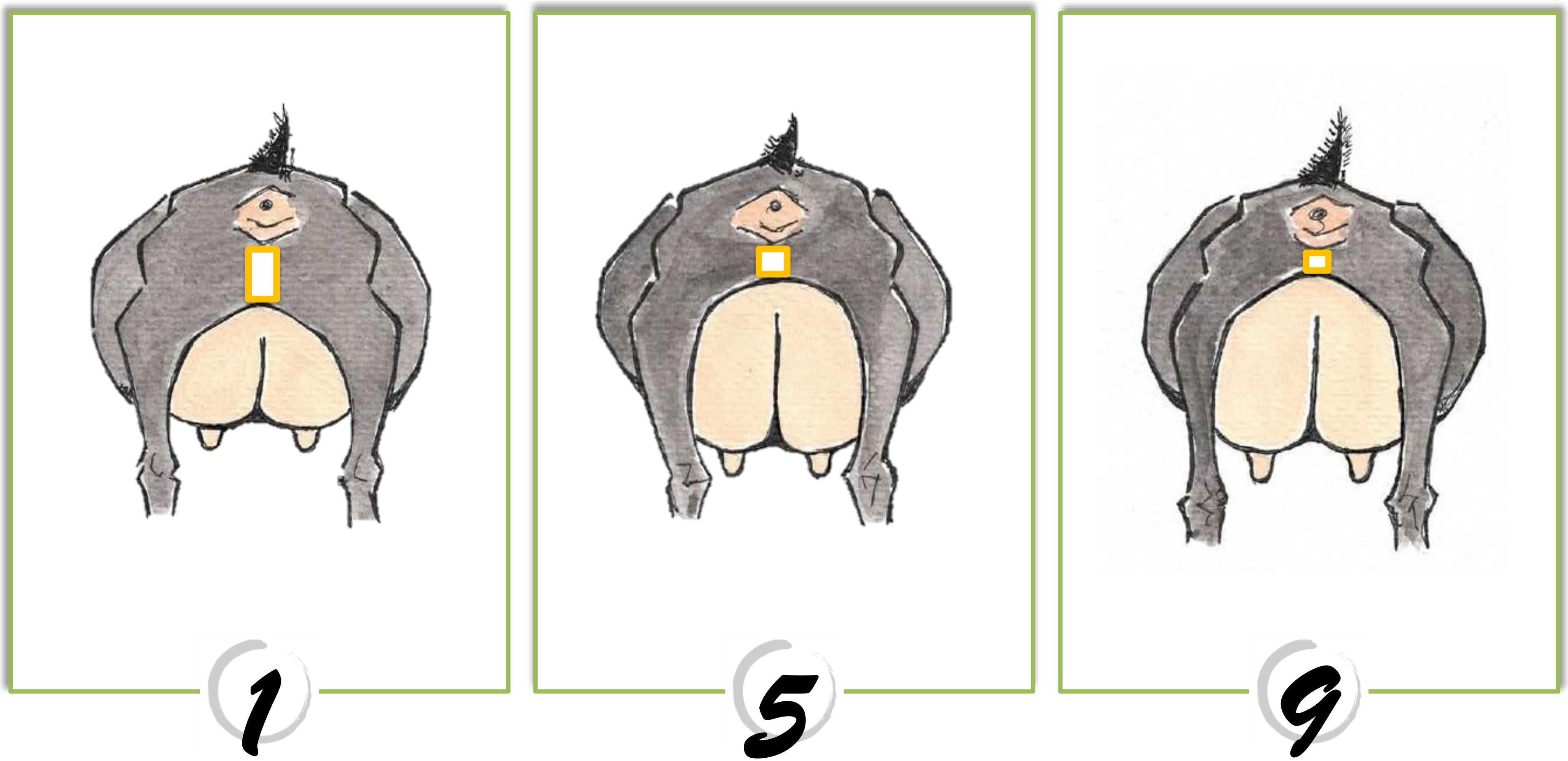

Measure unit	Score								
	1	2	3	4	5	6	7	8	9
cm	11	10	9	8	7	6	5	4	3

The scale translation from LAS scores to traditional zoometric measurements in centimeters, as well as optimal values following the premises described in the breed standard for both phenotype collection methods, are provided below the depictions.

Figure S10: Graphical depictions of the scale for median suspensor ligament in Murciano-Granadina does for dairy purpose-related zoometric assessment.

Median suspensor ligament

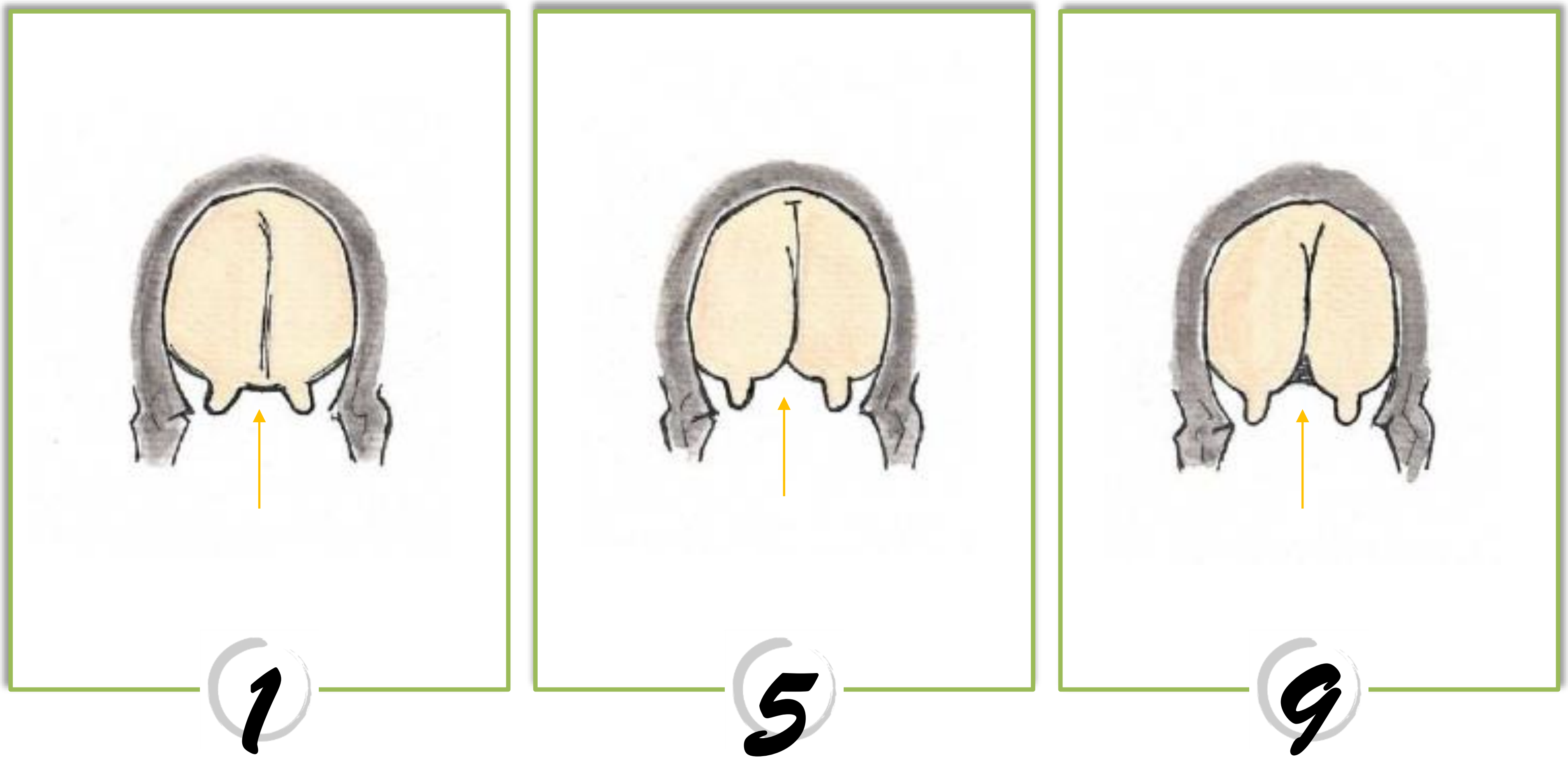

Measure unit	Score								
	1	2	3	4	5	6	7	8	9
cm	1	2	3	4	5	6	7	8	9

The scale translation from LAS scores to traditional zoometric measurements in centimeters, as well as optimal values following the premises described in the breed standard for both phenotype collection methods, are provided below the depictions.

Figure S11: Graphical depictions of the scale for udder width in Murciano-Granadina does for dairy purpose-related zoometric assessment.

Udder width

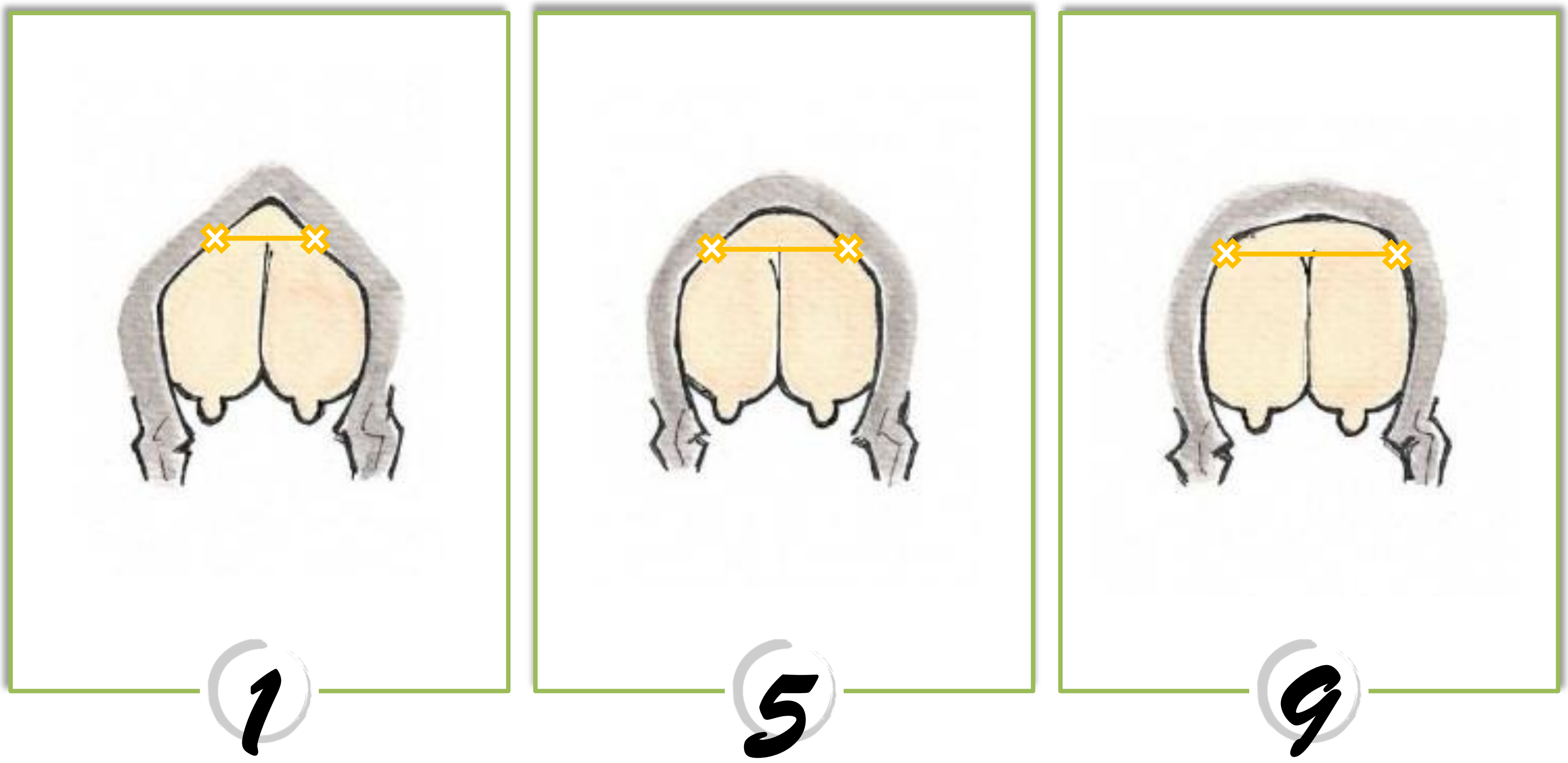

Measure unit	Score								
	1	2	3	4	5	6	7	8	9
cm	3	4	5	6	7	8	9	10	11

The scale translation from LAS scores to traditional zoometric measurements in centimeters, as well as optimal values following the premises described in the breed standard for both phenotype collection methods, are provided below the depictions.

Figure S12: Graphical depictions of the scale for udder width in Murciano-Granadina does for dairy purpose-related zoometric assessment.

Udder depth

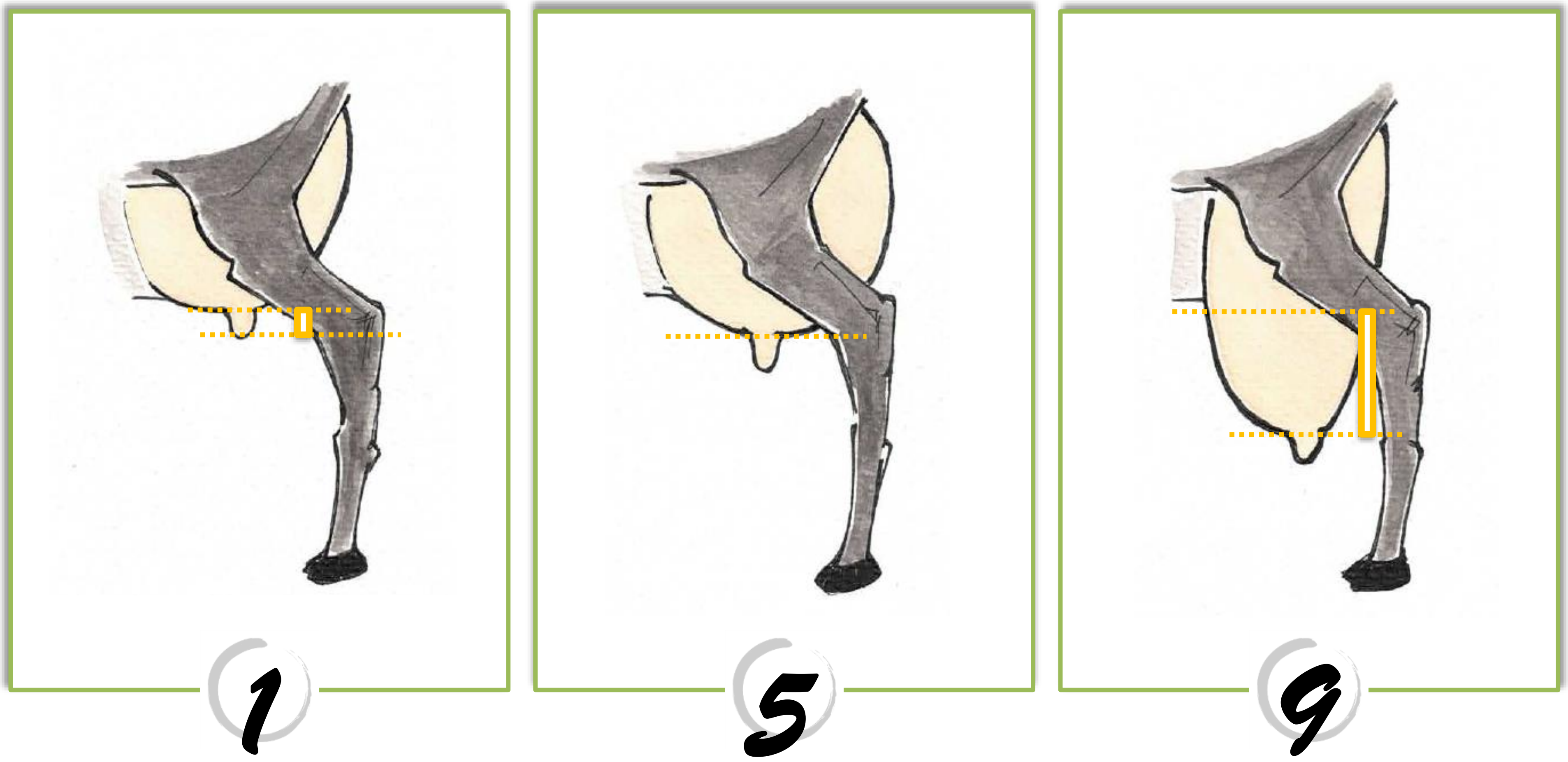

Measure unit	Score								
	1	2	3	4	5	6	7	8	9
cm	-10	-7,5	-5	-2,5	0	2,5	5	7,5	10

The scale translation from LAS scores to traditional zoometric measurements in centimeters, as well as optimal values following the premises described in the breed standard for both phenotype collection methods, are provided below the depictions.

Figure S13: Graphical depictions of the scale for nipple placement in Murciano-Granadina does for dairy purpose-related zoometric assessment.

Nipple placement

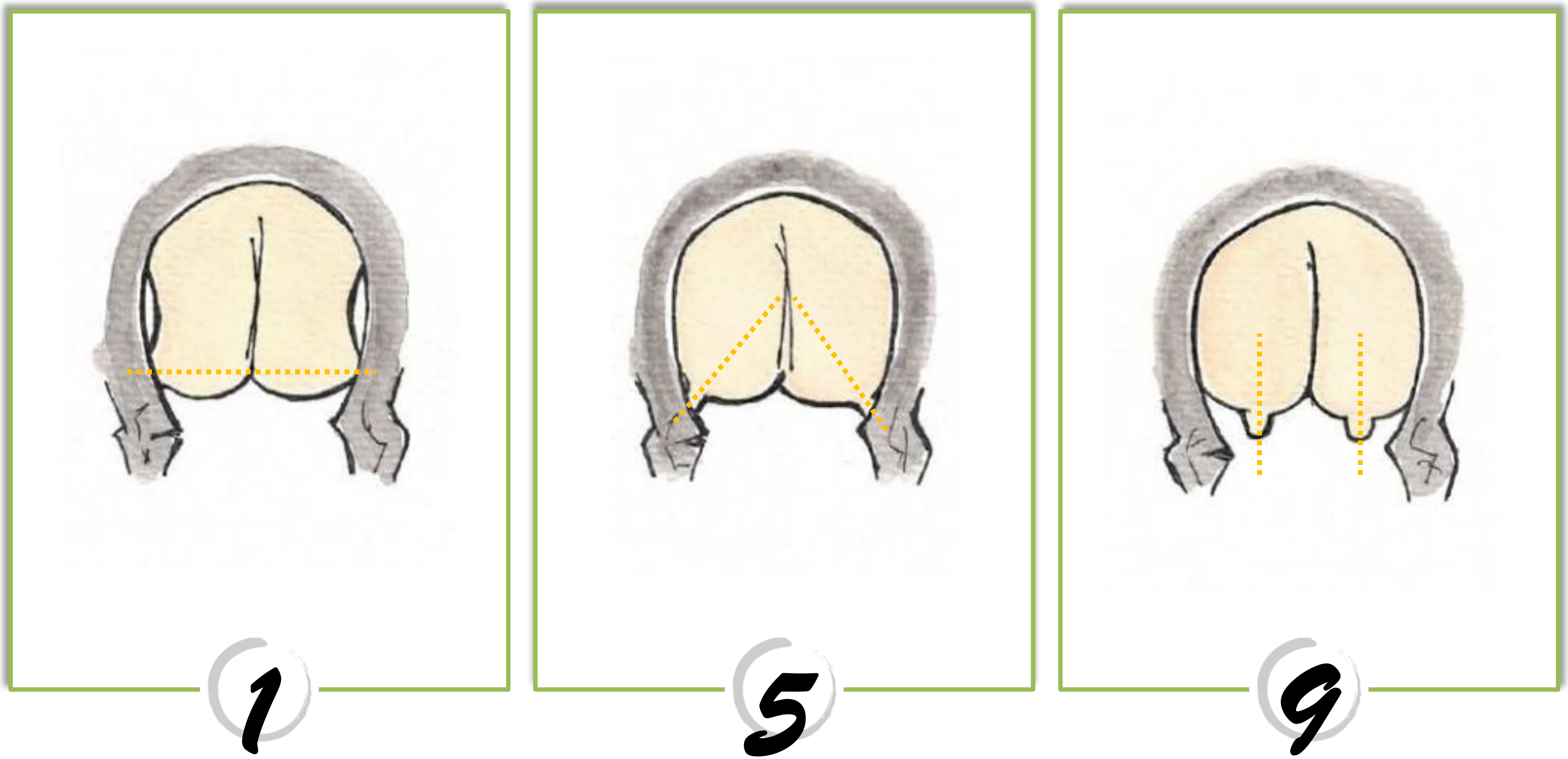

Score		
1	5	9
90º	45º	0º

The scale translation from LAS scores to traditional zoometric measurements in centimeters, as well as optimal values following the premises described in the breed standard for both phenotype collection methods, are provided below the depictions.

Figure S14: Graphical depictions of the scale for nipple diameter in Murciano-Granadina does for dairy purpose-related zoometric assessment.

Nipple diameter

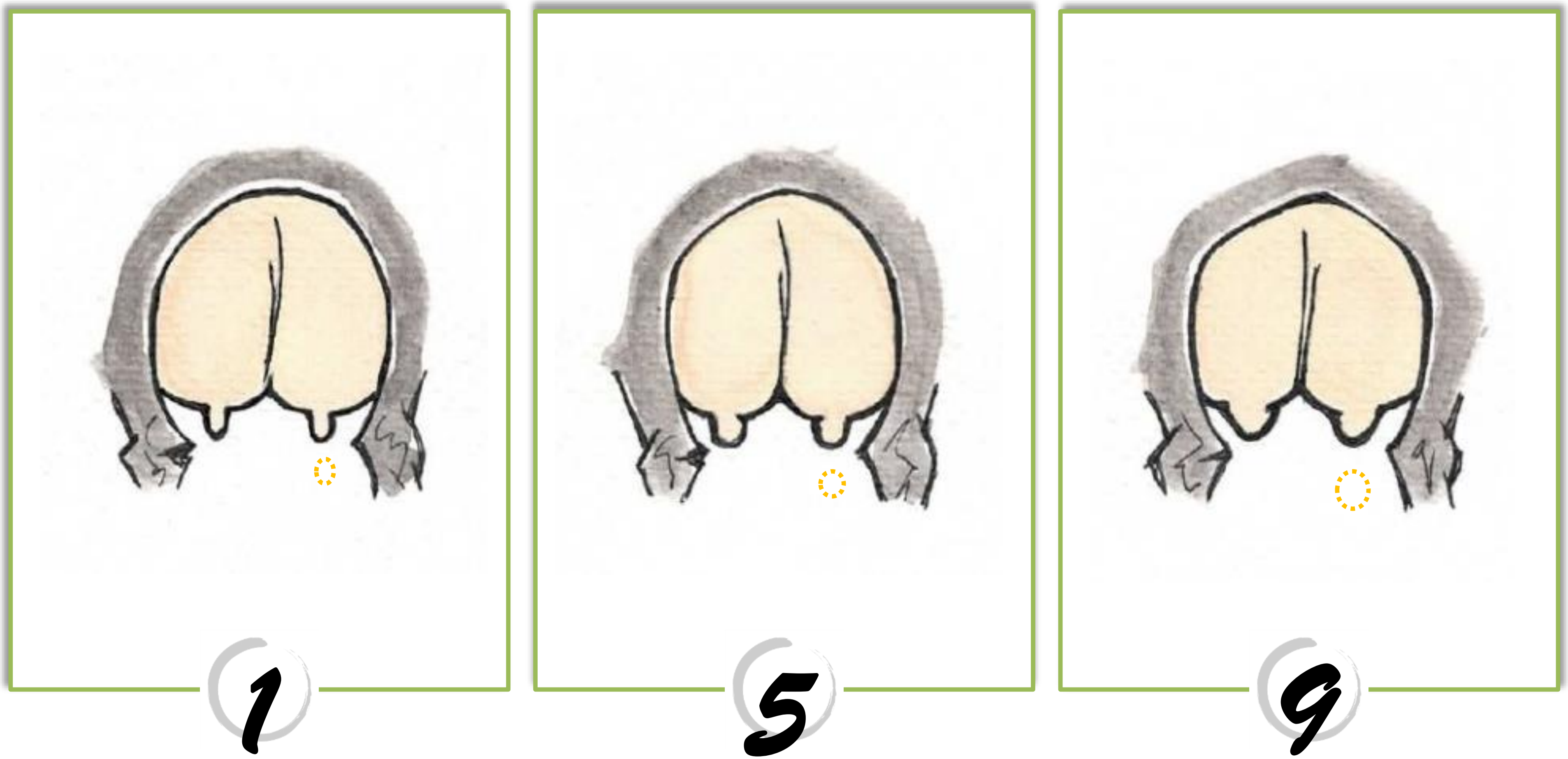

Measure unit	Score								
	1	2	3	4	5	6	7	8	9
cm	0,5	1	1,5	2	2,5	3	3,5	4	4,5

The scale translation from LAS scores to traditional zoometric measurements in centimeters, as well as optimal values following the premises described in the breed standard for both phenotype collection methods, are provided below the depictions.

Figure S15: Graphical depictions of the scale for rear legs, rear view in Murciano-Granadina does for dairy purpose-related zoometric assessment.

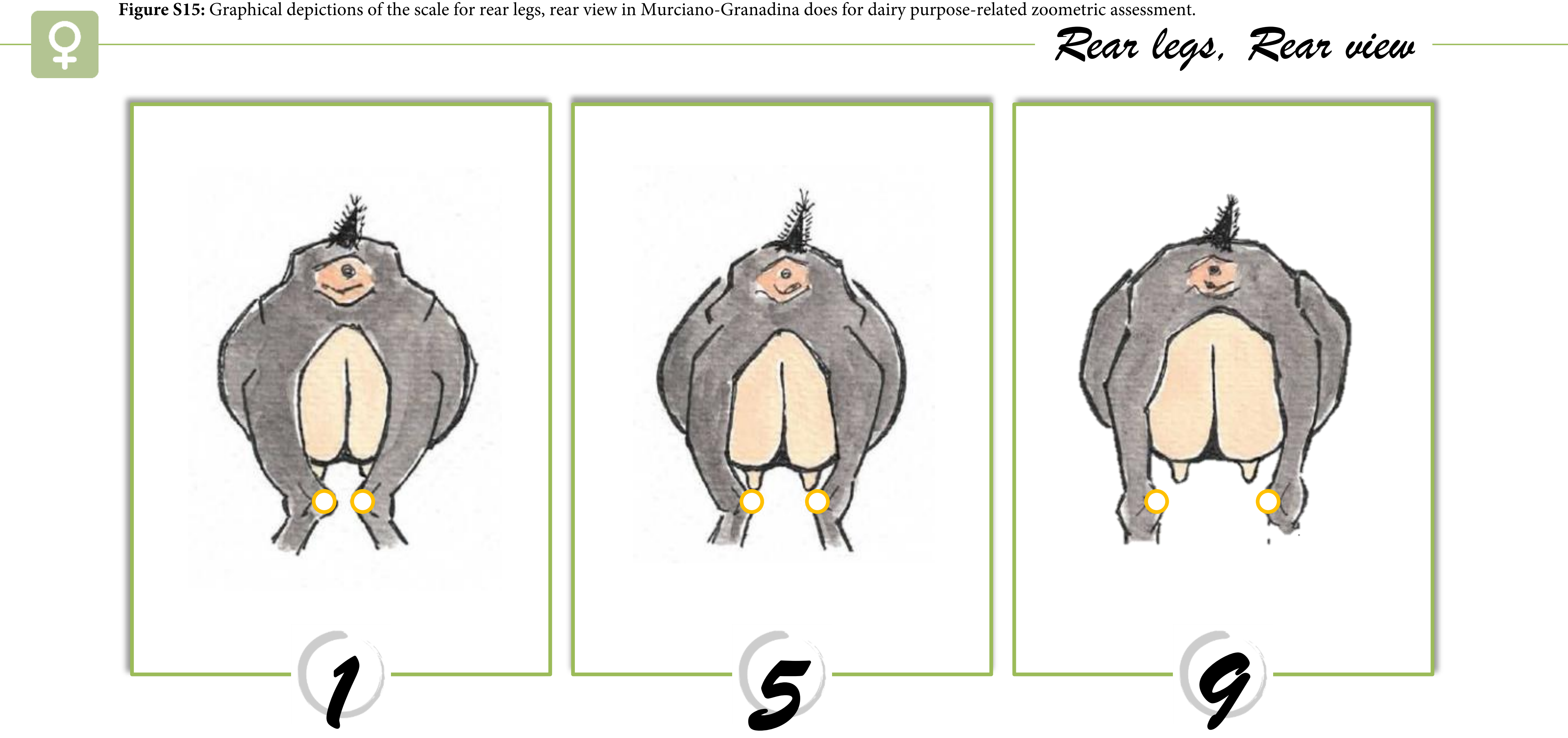

Score		
1	5	9
Very closed hocks	Slightly closed hocks	Parallel and separated hocks

The scale translation from LAS scores to traditional zoometric measurements in centimeters, as well as optimal values following the premises described in the breed standard for both phenotype collection methods, are provided below the depictions.

Figure S16: Graphical depictions of the scale for rear legs, side view in Murciano-Granadina does for dairy purpose-related zoometric assessment.

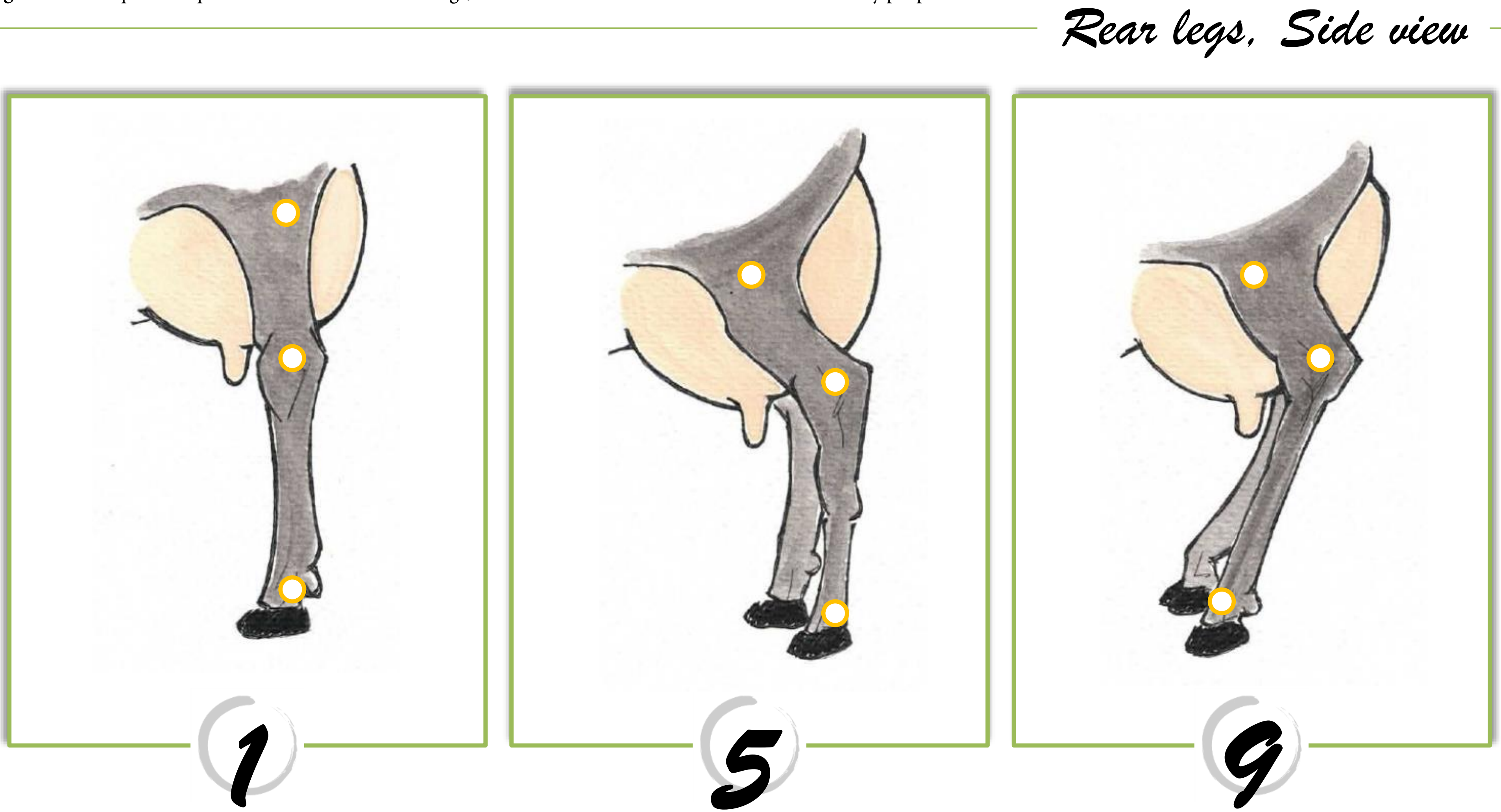

Score		
1	5	9
Straight	Desirable curvature	Very curved

The scale translation from LAS scores to traditional zoometric measurements in centimeters, as well as optimal values following the premises described in the breed standard for both phenotype collection methods, are provided below the depictions.

Figure S17: Graphical depictions of the scale for mobility in Murciano-Granadina does for dairy purpose-related zoometric assessment.

Mobility

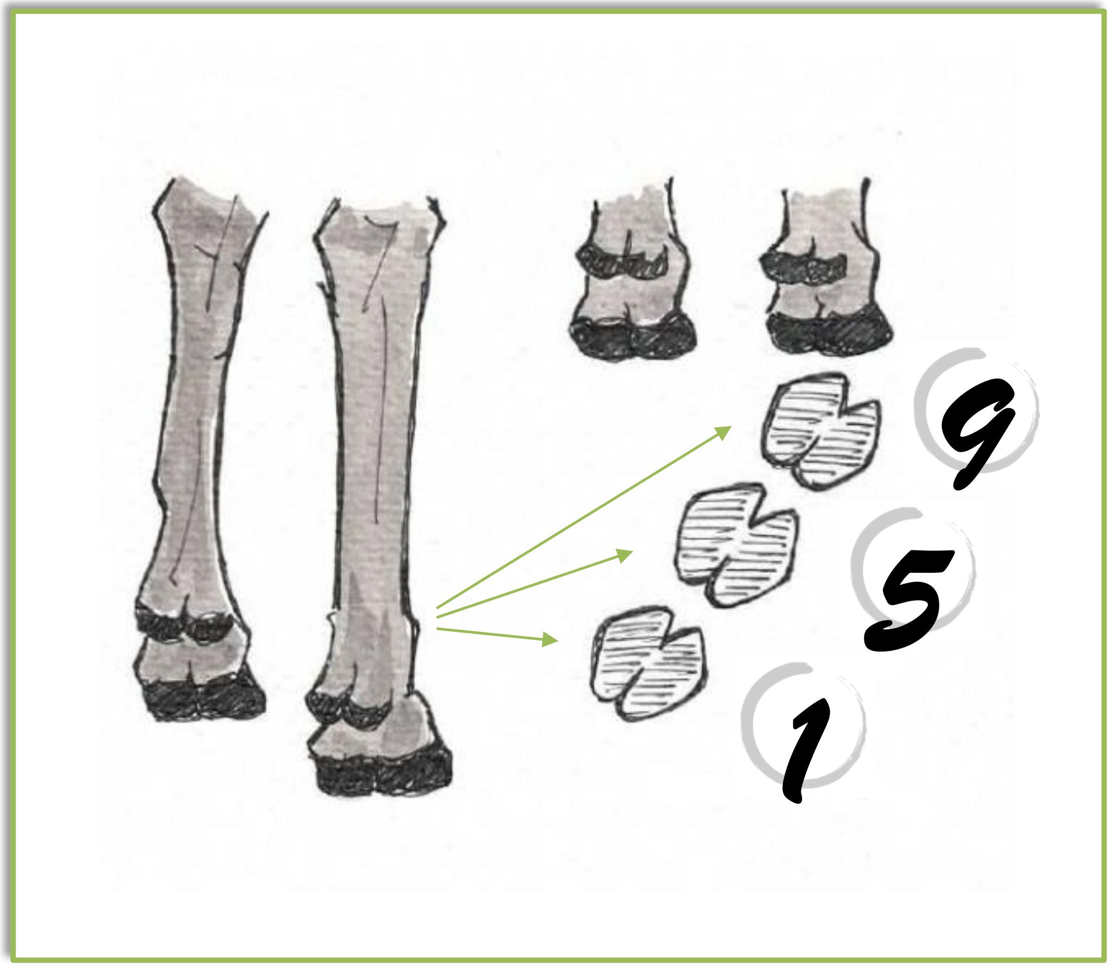

1	Very bad mobility due to skeleton structure
2	Severe ab/adduction. Unequal step. Short stride
3	Ab/adduction. Unequal step
4	Slight ab/adduction. Uniform step. Short stride
5	Very slight ab/adduction. Uniform step. Intermediate stride
6	Straight and uniform step. Intermediate stride
7	Straight and uniform step. Long stride
8	Straight and uniform step. Long and strong stride
9	Straight and uniform step. Too long and strong stride

The scale translation from LAS scores to traditional zoometric measurements in centimeters, as well as optimal values following the premises described in the breed standard for both phenotype collection methods, are provided below the depictions.

Figure S18: Graphical depictions of the scale for stature in Murciano-Granadina bucks for dairy purpose-related zoometric assessment.

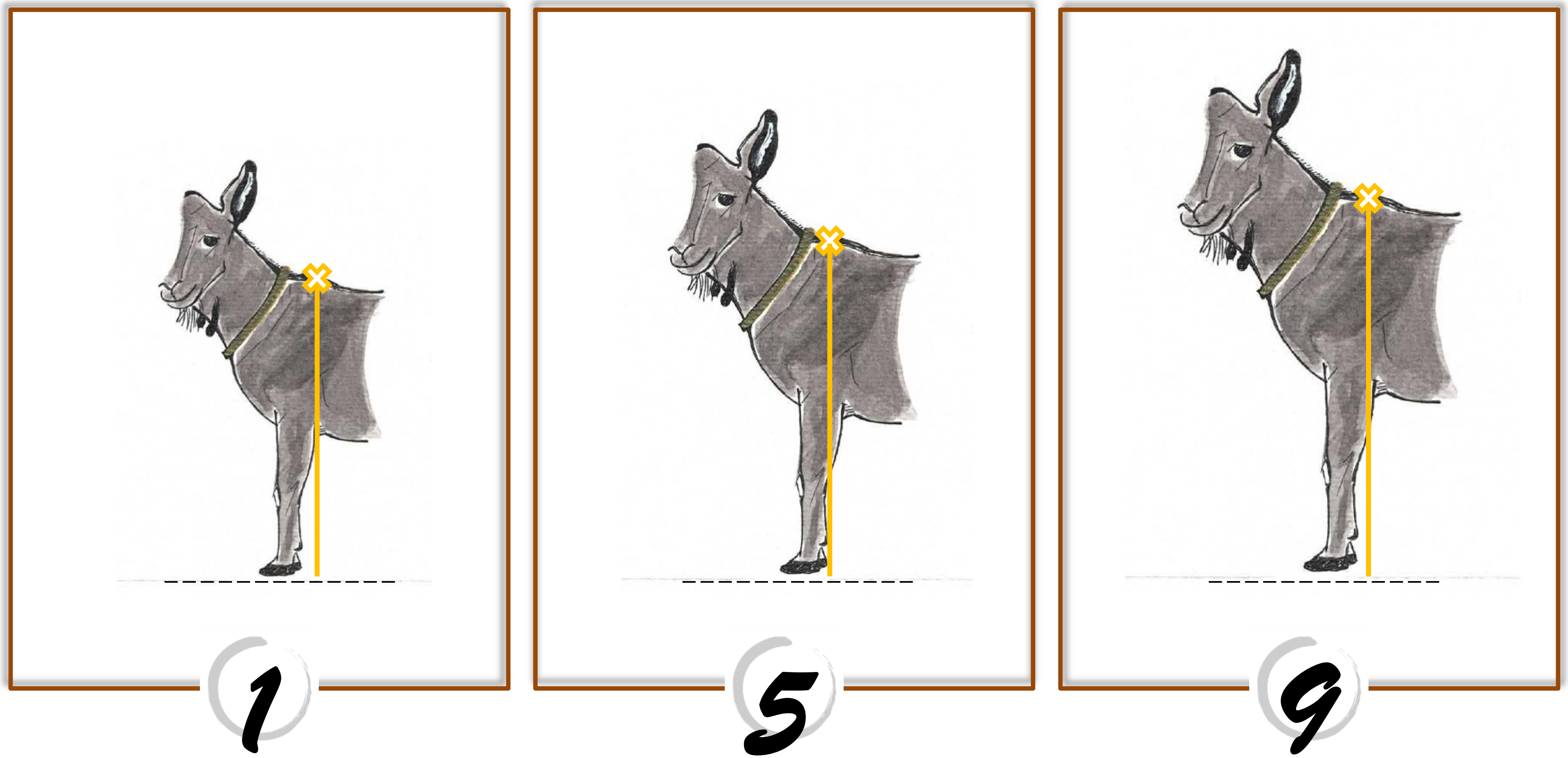

Measure unit	Score								
	1	2	3	4	5	6	7	8	9
cm	68	71	74	77	80	83	86	86	92

The scale translation from LAS scores to traditional zoometric measurements in centimeters, as well as optimal values following the premises described in the breed standard for both phenotype collection methods, are provided below the depictions.

Figure S19: Graphical depictions of the scale for chest width in Murciano-Granadina bucks for dairy purpose-related zoometric assessment.

Chest width

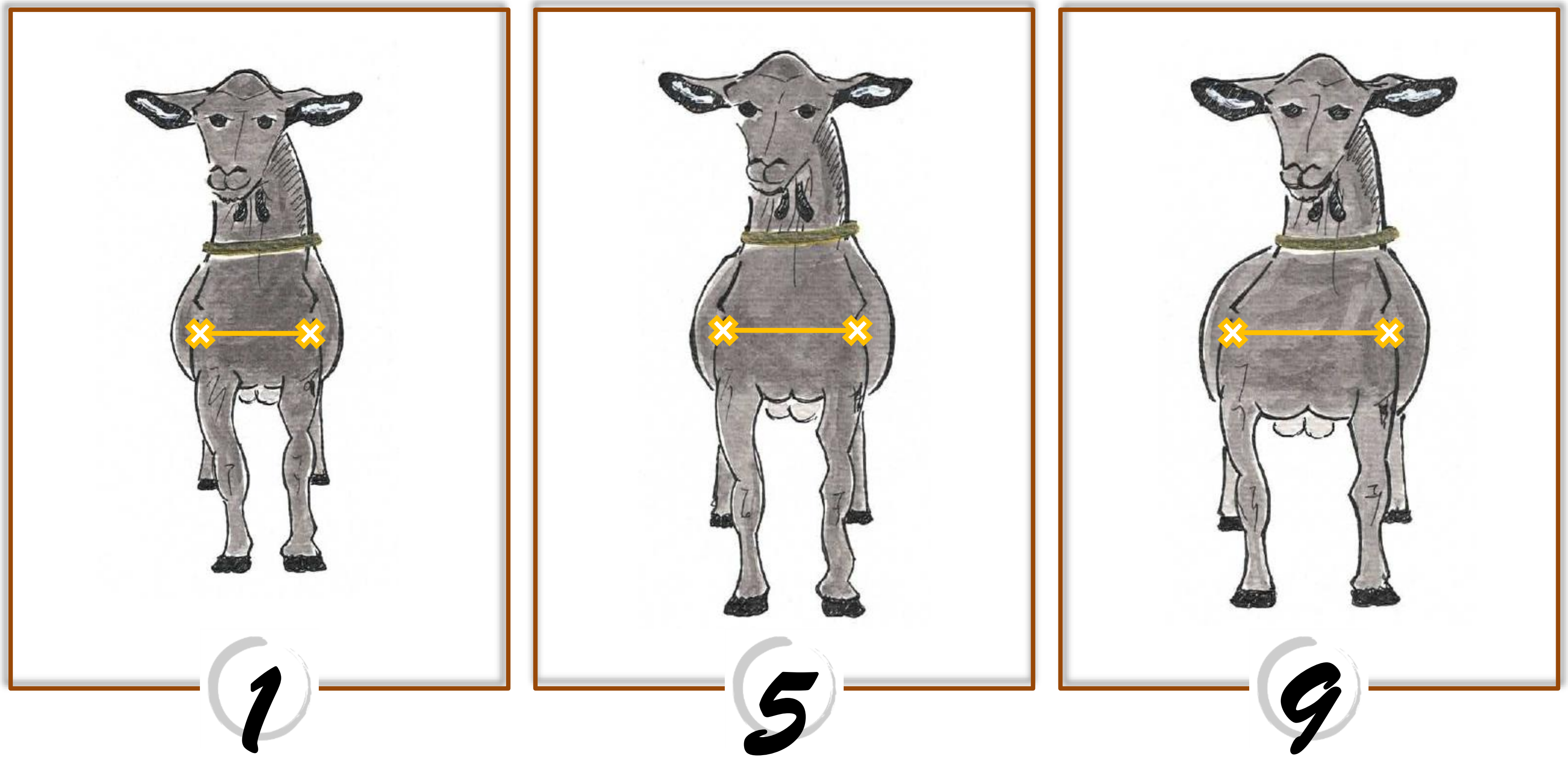

Measure unit	Score								
	1	2	3	4	5	6	7	8	9
cm	15	17	19	21	23	25	27	29	31

The scale translation from LAS scores to traditional zoometric measurements in centimeters, as well as optimal values following the premises described in the breed standard for both phenotype collection methods, are provided below the depictions.

Figure S20: Graphical depictions of the scale for body depth in Murciano-Granadina bucks for dairy purpose-related zoometric assessment.

Body depth

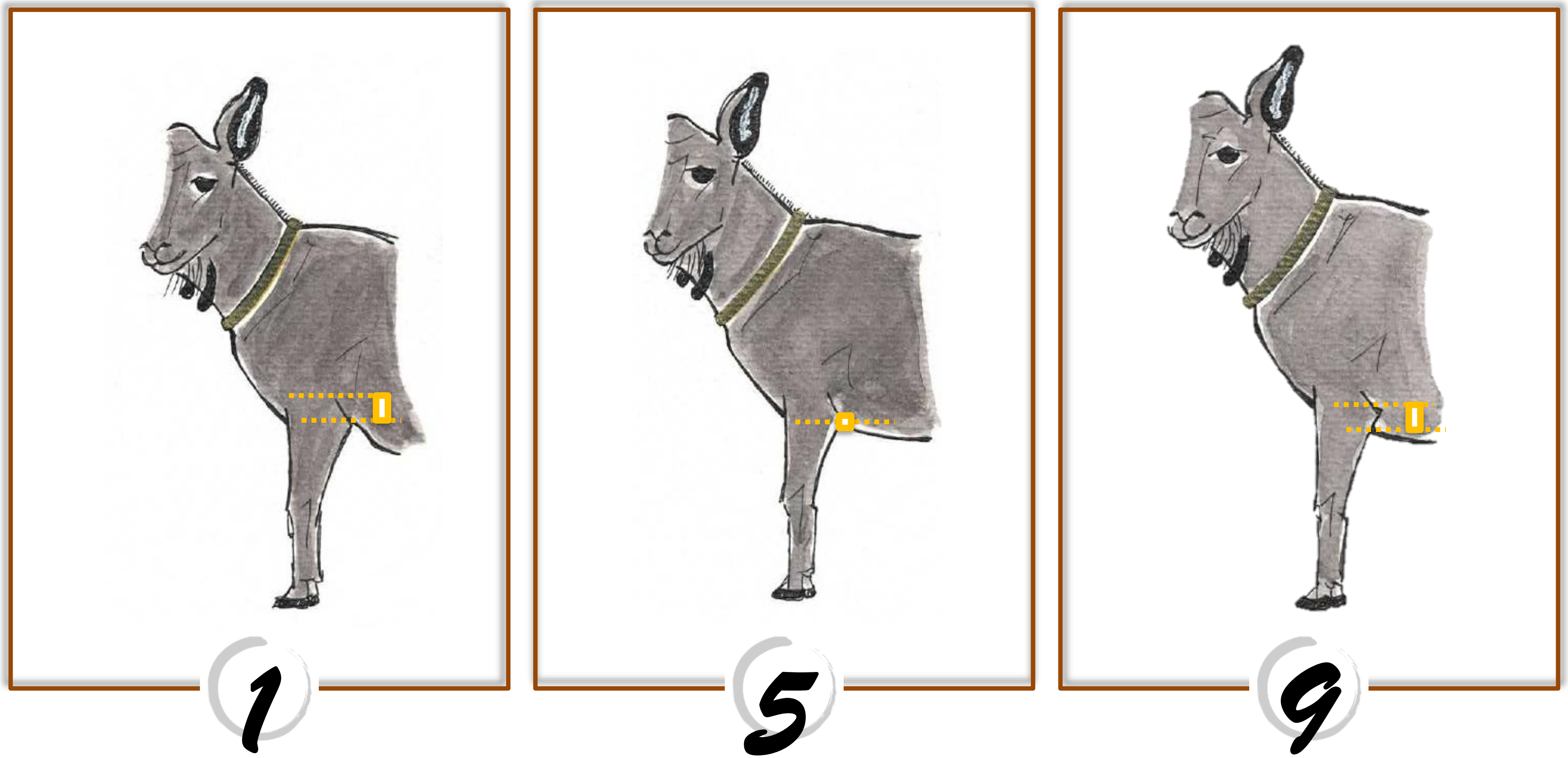

Score		
1	5	9
Shallow	Intermediate	Extremely deep

The scale translation from LAS scores to traditional zoometric measurements in centimeters, as well as optimal values following the premises described in the breed standard for both phenotype collection methods, are provided below the depictions.

Figure S21: Graphical depictions of the scale for rump width in Murciano-Granadina bucks for dairy purpose-related zoometric assessment.

Rump width

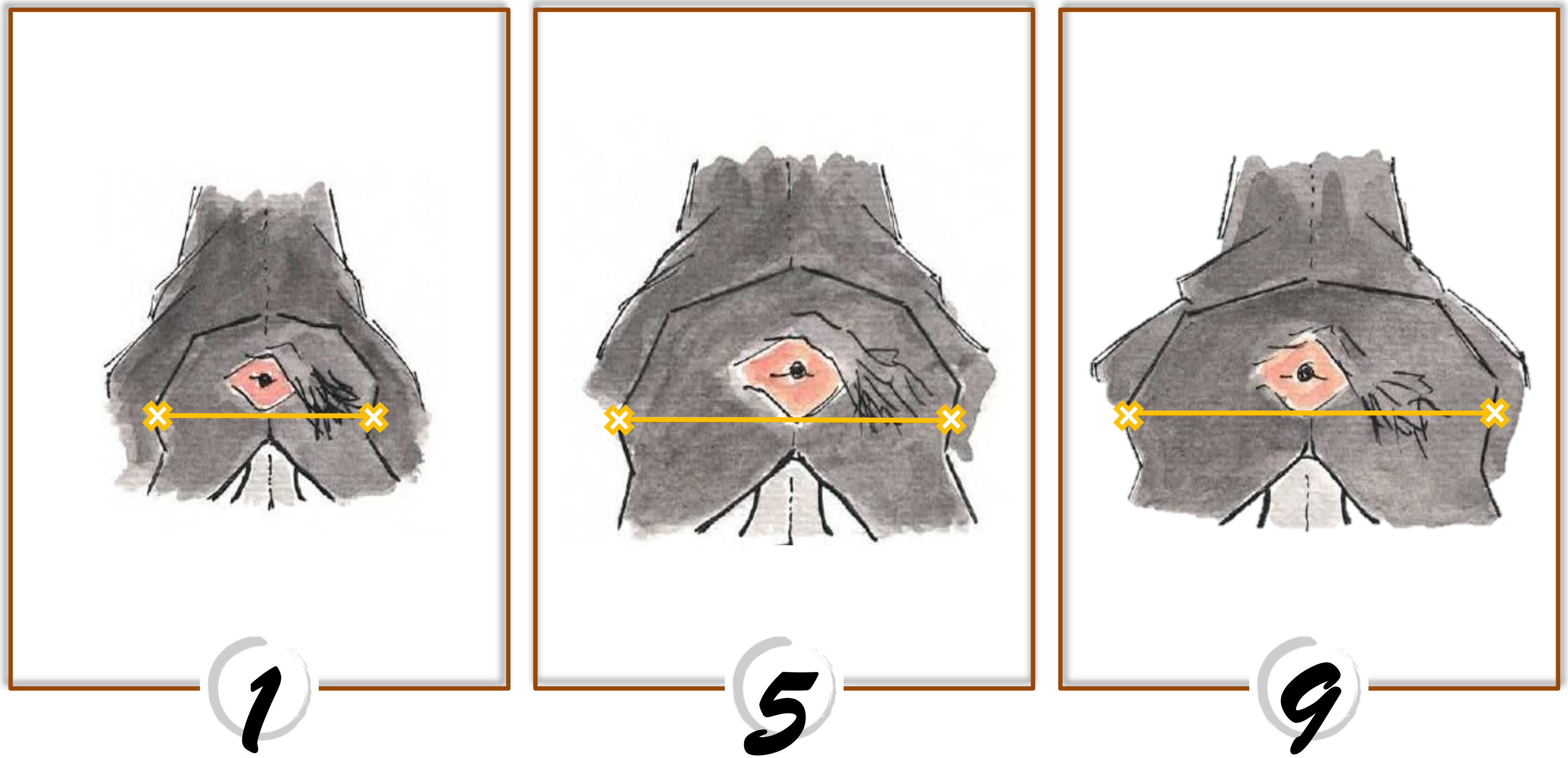

Measure unit	Score								
	1	2	3	4	5	6	7	8	9
cm	14	15	16	17	18	19	20	21	22

The scale translation from LAS scores to traditional zoometric measurements in centimeters, as well as optimal values following the premises described in the breed standard for both phenotype collection methods, are provided below the depictions.

Figure S22: Graphical depictions of the scale for rump angle in Murciano-Granadina bucks for dairy purpose-related zoometric assessment.

Rump angle

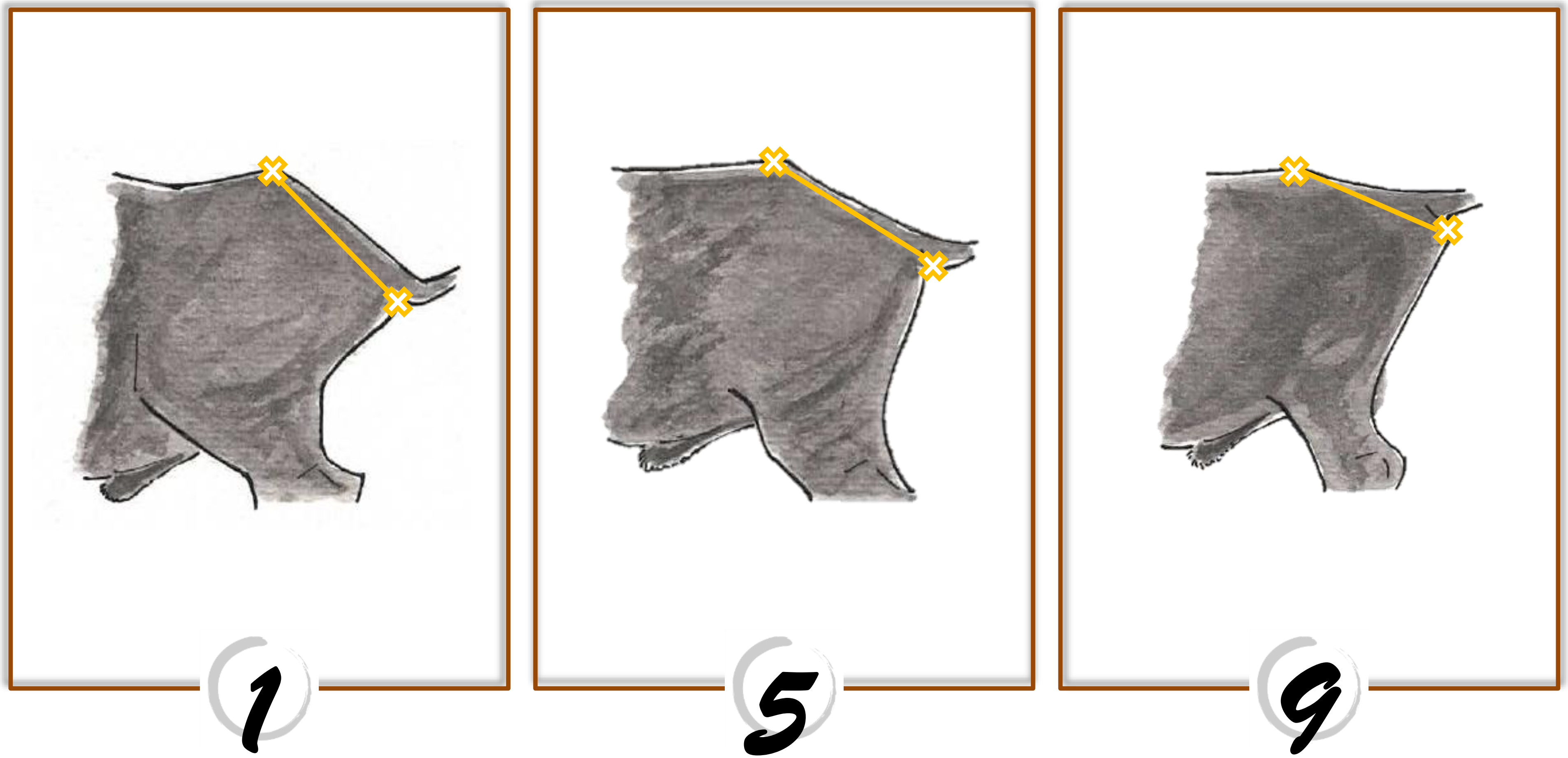

Measure unit	Score								
	1	2	3	4	5	6	7	8	9
cm	55	52	49	46	43	40	37	34	31

The scale translation from LAS scores to traditional zoometric measurements in centimeters, as well as optimal values following the premises described in the breed standard for both phenotype collection methods, are provided below the depictions.

Figure S23: Graphical depictions of the scale for angulosity in Murciano-Granadina bucks for dairy purpose-related zoometric assessment.

Angulosity

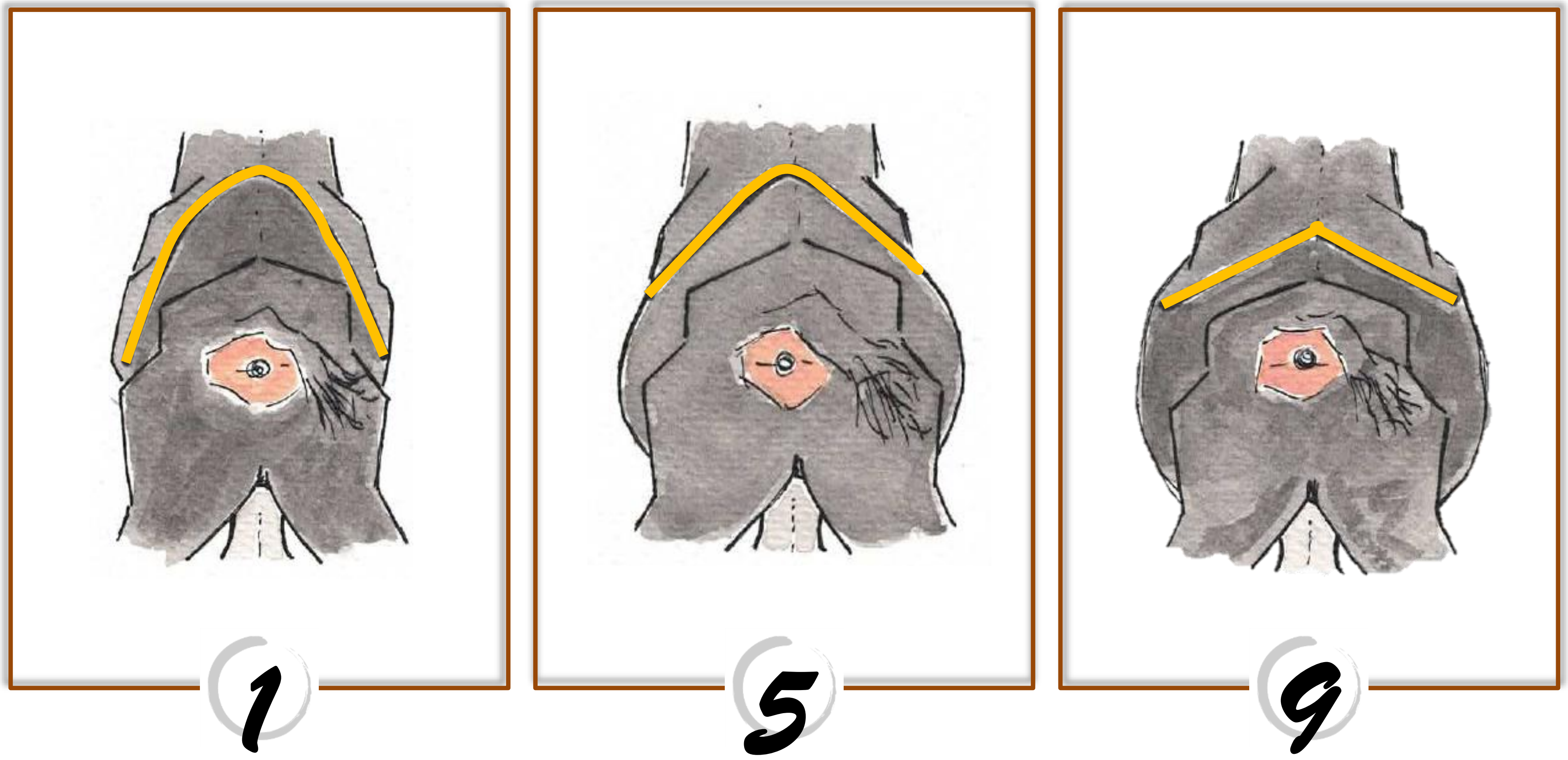

Score		
1	5	9
Rough extremity	Intermediate	Angulous extremity

The scale translation from LAS scores to traditional zoometric measurements in centimeters, as well as optimal values following the premises described in the breed standard for both phenotype collection methods, are provided below the depictions.

Figure S24: Graphical depictions of the scale for bone quality in Murciano-Granadina bucks for dairy purpose-related zoometric assessment.

Bone quality

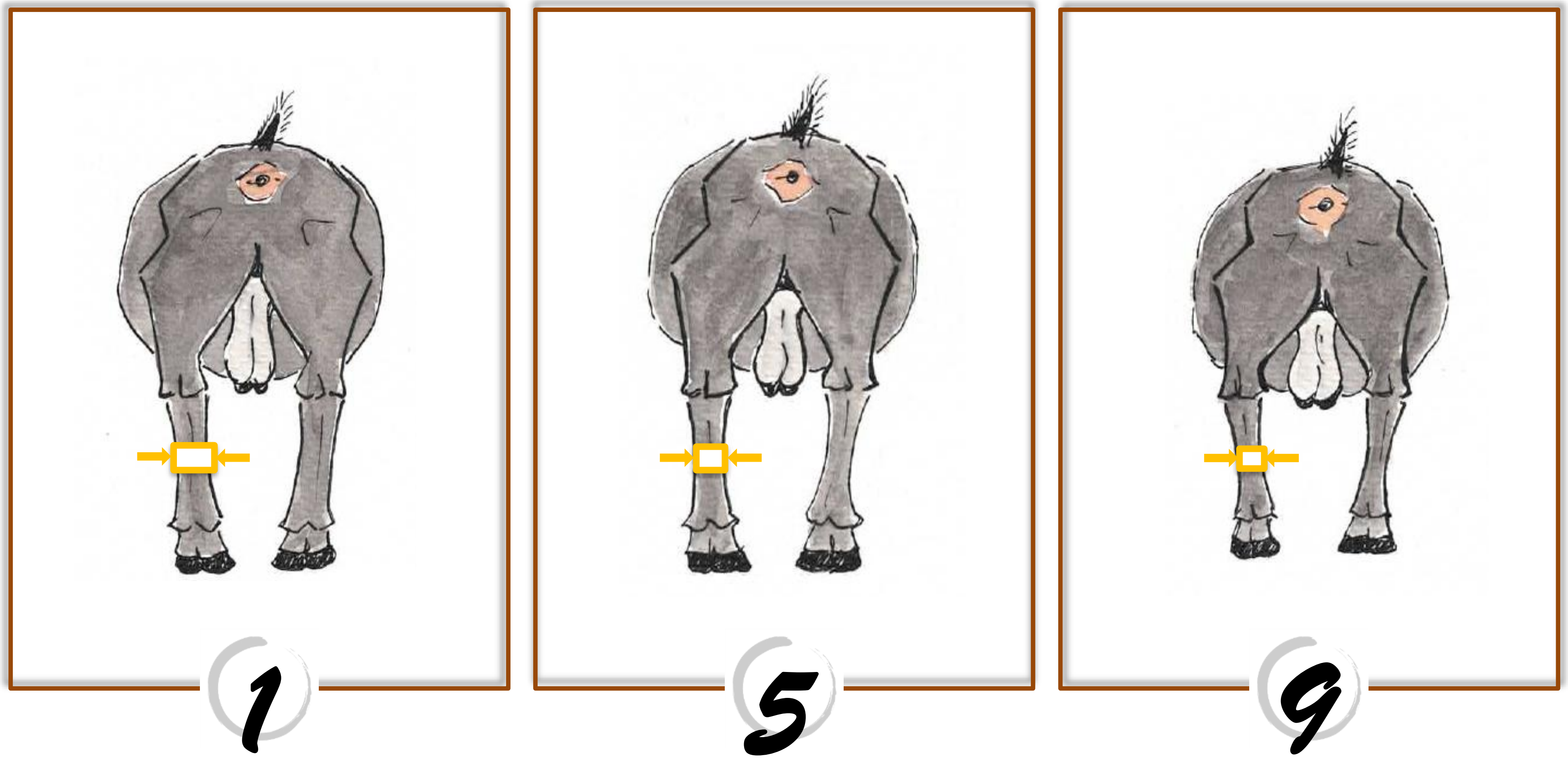

Score		
1	5	9
Round and rough bones	Intermediate	Flat and neat bones

The scale translation from LAS scores to traditional zoometric measurements in centimeters, as well as optimal values following the premises described in the breed standard for both phenotype collection methods, are provided below the depictions.

Figure S25: Graphical depictions of the scale for rear legs, rear view in Murciano-Granadina bucks for dairy purpose-related zoometric assessment.

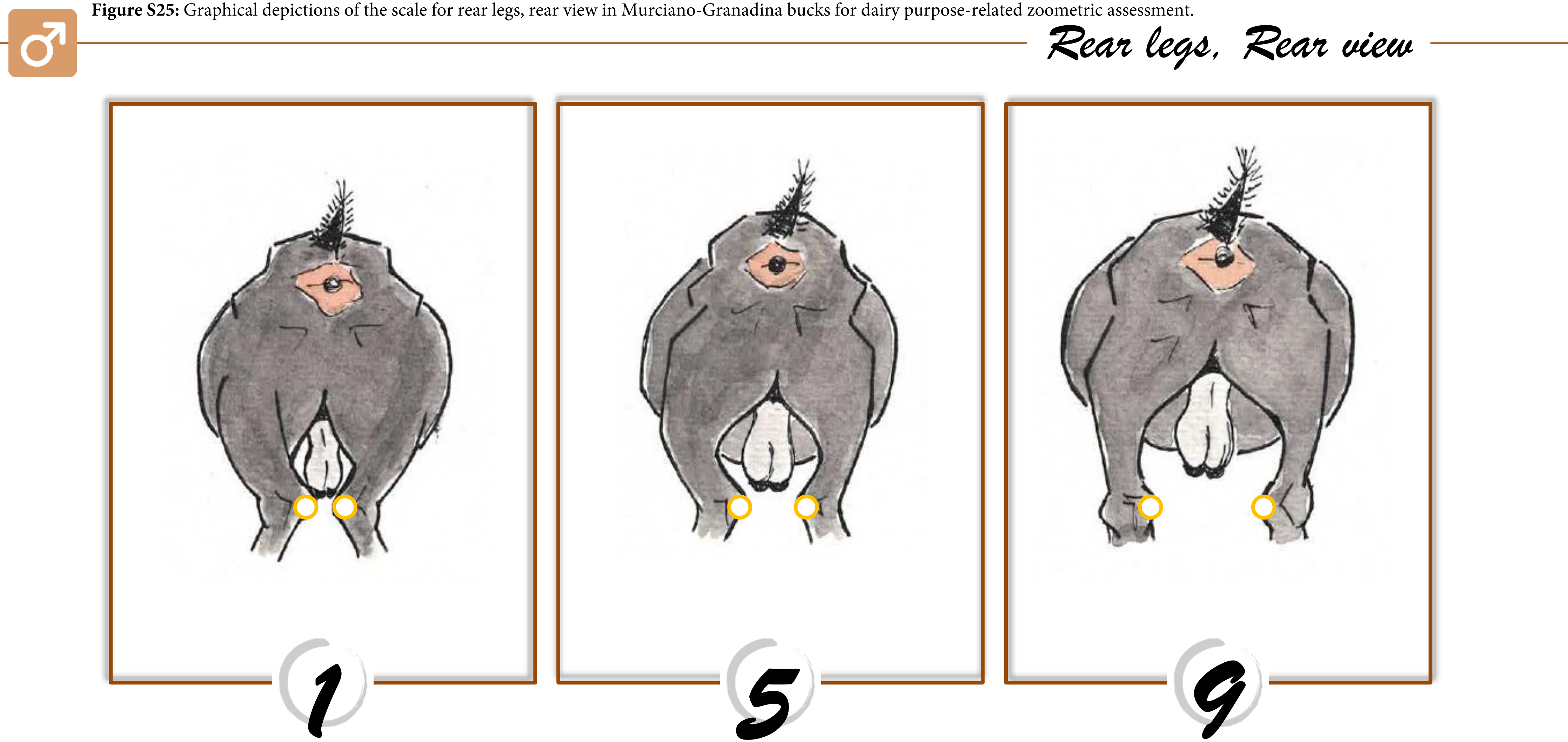

Score		
1	5	9
Very closed hocks	Slightly closed hocks	Parallel and separated hocks

The scale translation from LAS scores to traditional zoometric measurements in centimeters, as well as optimal values following the premises described in the breed standard for both phenotype collection methods, are provided below the depictions.

Figure S26: Graphical depictions of the scale for rear legs, side view in Murciano-Granadina bucks for dairy purpose-related zoometric assessment.

Rear legs, Side view

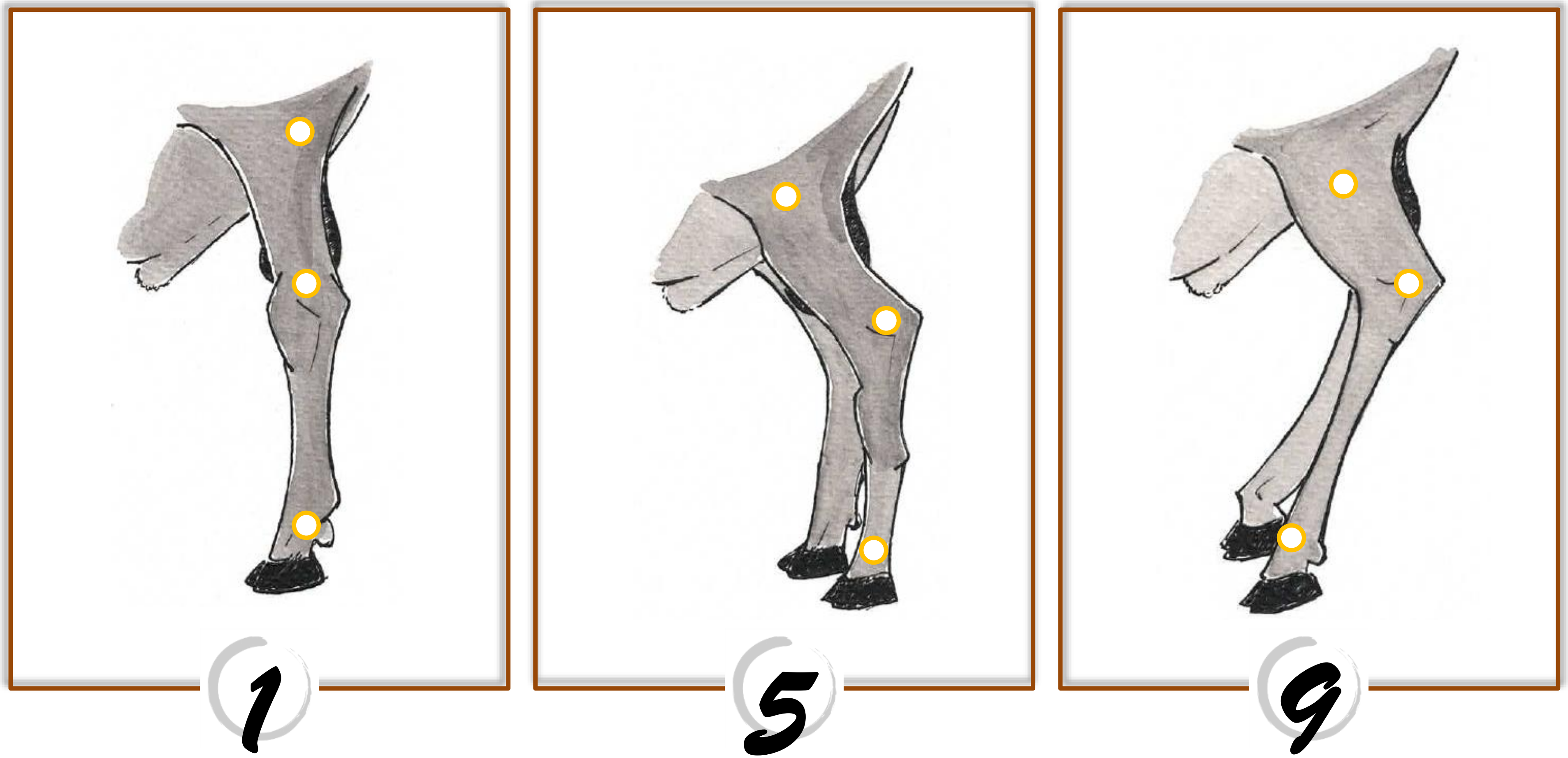

Score		
1	5	9
Straight	Desirable curvature	Very curved

The scale translation from LAS scores to traditional zoometric measurements in centimeters, as well as optimal values following the premises described in the breed standard for both phenotype collection methods, are provided below the depictions.

Figure S27: Graphical depictions of the scale for mobility in Murciano-Granadina bucks for dairy purpose-related zoometric assessment.

Mobility

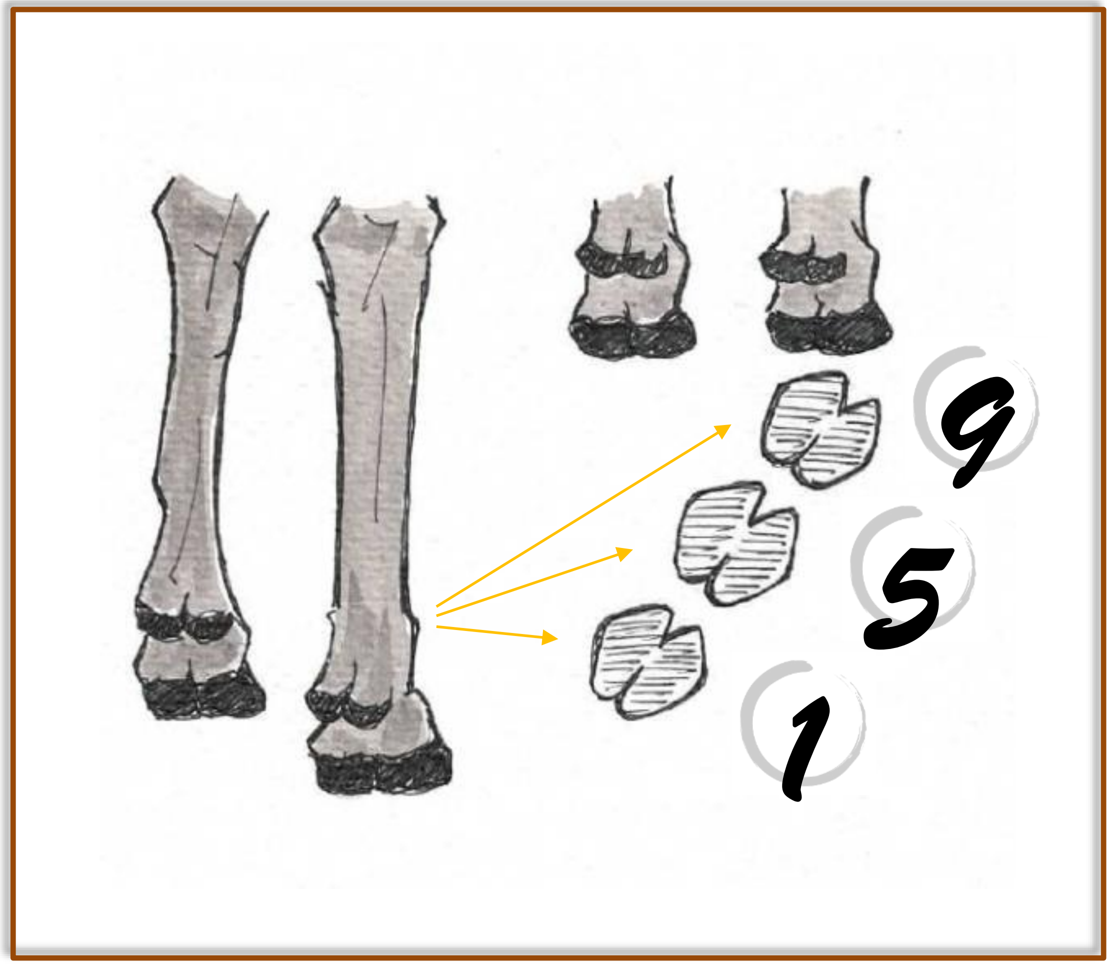

1	Very bad mobility due to skeleton structure
2	Severe ab/adduction. Unequal step. Short stride
3	Ab/adduction. Unequal step
4	Slight ab/adduction. Uniform step. Short stride
5	Very slight ab/adduction. Uniform step. Intermediate stride
6	Straight and uniform step. Intermediate stride
7	Straight and uniform step. Long stride
8	Straight and uniform step. Long and strong stride
9	Straight and uniform step. Too long and strong stride

The scale translation from LAS scores to traditional zoometric measurements in centimeters, as well as optimal values following the premises described in the breed standard for both phenotype collection methods, are provided below the depictions.
