# Supplementary material for: Analysis of the Genetic Parameters for Dairy Linear Appraisal and Zoometric Traits: A Tool to Enhance the Applicability of Murciano-Granadina Goats Major Areas Evaluation System
Source: Animals (Basel). 2023 Mar 21;13(6):1114. doi: 10.3390/ani13061114 (PMC10044043; doi:10.3390/ani13061114)
Supplement: Supplementary file 1 [file animals-13-01114-s001.zip › Table S5.docx]

**Table S5.** Gain (+)/Loss (-) in heritability and increase (+)/decrease (-) in heritability standard errors for zoometric and LAS traits in Murciano-Granadina goats and bucks from 2011 to 2021.

| Zoometric/LAS Trait | Gain (+)/Loss (-) in h^2^ from 2011 to 2021 | Increase (+)/Decrease (-) in h^2^ SE from 2011 to 2021 |
| --- | --- | --- |
| Stature (Height to withers) | 0.21 | -0.11 |
| Chest Width | 0.01 | -0.11 |
| Body Depth | 0.10 | 0.00 |
| Rump Width | 0.05 | -0.11 |
| Rump Angle | 0.17 | 0.00 |
| Angulosity | 0.07 | -0.09 |
| Bone Quality | 0.31 | 0.00 |
| Anterior insertion | 0.09 | -0.08 |
| Rear Insertion Height | 0.10 | -0.09 |
| Median Suspensor Ligament | 0.21 | -0.09 |
| Udder width | 0.10 | 0.00 |
| Udder Depth | 0.12 | -0.10 |
| Nipple placement | 0.07 | -0.12 |
| Nipple Diameter | 0.14 | -0.11 |
| Rear Legs Rear View | 0.05 | -0.09 |
| Rear Legs Side View | 0.09 | 0.00 |
| Mobility | -0.05 | -0.10 |
